# Transcriptome and metabolome analysis revealed the dynamic change of bioactive compounds of Fructus Ligustri Lucidi

**DOI:** 10.1186/s12870-024-05096-3

**Published:** 2024-06-03

**Authors:** Peina Zhou, Jingjie Dang, Zheng Jiang, Shilin Dai, Cheng Qu, Qinan Wu

**Affiliations:** 1 College of Pharmacy, Nanjing University of Chinese Medicine, Nanjing, 210023 China; 2 Collaborative Innovation Center of Chinese Medicinal Resources Industrialization, Nanjing, 210023 China; 3 National and Local Collaborative Engineering Center of Chinese Medicinal Resources Industrialization and Formulae Innovative Medicine, Nanjing, 210023 China

**Keywords:** Fructus Ligustri Lucidi, Metabolome, Transcriptome, Secoiridoid biosynthesis, Salidroside biosynthesis

## Abstract

**Background:**

The Fructus Ligustri Lucidi, the fruit of *Ligustrum lucidum*, contains a variety of bioactive compounds, such as flavonoids, triterpenoids, and secoiridoids. The proportions of these compounds vary greatly during the different fruit development periods of Fructus Ligustri Lucidi. However, a clear understanding of how the proportions of the compounds and their regulatory biosynthetic mechanisms change across the different fruit development periods of Fructus Ligustri Lucidi is still lacking.

**Results:**

In this study, metabolite profiling and transcriptome analysis of six fruit development periods (45 DAF, 75 DAF, 112 DAF, 135 DAF, 170 DAF, and 195 DAF) were performed. Seventy compounds were tentatively identified, of which secoiridoids were the most abundant. Eleven identified compounds were quantified by high performance liquid chromatography. A total of 103,058 unigenes were obtained from six periods of Fructus Ligustri Lucidi. Furthermore, candidate genes involved in triterpenoids, phenylethanols, and oleoside-type secoiridoid biosynthesis were identified and analyzed. The in vitro enzyme activities of nine glycosyltransferases involved in salidroside biosynthesis revealed that they can catalyze trysol and hydroxytyrosol to salidroside and hydroxylsalidroside.

**Conclusions:**

These results provide valuable information to clarify the profile and molecular regulatory mechanisms of metabolite biosynthesis, and also in optimizing the harvest time of this fruit.

**Supplementary Information:**

The online version contains supplementary material available at 10.1186/s12870-024-05096-3.

## Background

Fructus Ligustri Lucidi (FLL) is the fruit of *Ligustrum lucidum*, which belongs to the *Oleaceae* family. *Ligustrum lucidum* is widely distributed in China, South Korea, India, and Australia and is often used for environmental greening. Its berry fruits, which are used as phytomedicine, are generally ovate, elliptic, or kidney-shaped, with a dark purple or grey-black color; they are usually harvested in winter (Fig. S1). In China, dried mature FLL is named Nvzhenzi, and it is used in traditional Chinese medicine (TCM) [1]. Modern chemical research has shown that triterpenes, phenylethanoid glycosides, secoiridoids, and flavonoids are the main secondary metabolites of FLL. These metabolites have a wide range of pharmacological effects, and are often used in clinical applications [2, 3]. FLL is therefore a promising nutritional food additive for humans. However, it is unclear which harvest time is best for optimizing the amount of active components in FLL, and thus its medicinal value. In addition, not enough is known about the biosynthetic pathways involved, or the dynamic changes of active components to choose the harvest time and utilization of FLL as a food additive.

Based on chemical and pharmacological studies of FLL, its main bioactive secoiridoids are oleuropein, nuezhenoside G13, and specneuzhenide [1–6]. There has been some researches related to secoiridoid biosynthesis in the *Oleaceae* family, specifically in olives (*Olea europaea* L.). Oleoside-type secoiridoids, which are found exclusively contained in the *Oleaceae* family and the genus *Caiophora* (*Loasaceae*), are some of the most frequently isolated and characterized biochemical components of FLL [4, 7]. Although the biosynthetic pathway of secoiridoids has not been elucidated in FLL, it has been examined in olives for oleoside-type secoiridoids such as oleuropein. That pathway can be described as follows. First, 7-deoxyloganic acid is hydroxylated; this hydroxylation is catalyzed by 7-deoxy-loganic acid 7-*epi*-hydroxylase (7eDLH), oleoside methyl ester synthase (OMES), and secoxyloganin synthase (SXS) to produce oleoside-11-methyl ester (OME). OME is then glycosylated by glycosyltransferase (GT) to produce 7-*β*-1-D-glucopyranosyl-11-methyl oleoside (OME-glu). Finally, OME-glu is then conjugated with phenolic components such as tyrosol and salidroside to produce oleoside-type secoiridoids [8]. This biosynthetic pathway may share similarities with the corresponding pathway in FLL. However, the biosynthesis of its major compounds in FLL, as well as transcriptional-level variations during FLL periods, have not been reported.

In this study, six fruit development periods of FLL including 45 days after flowering (DAF), 75 DAF, 112 DAF, 135 DAF, 170 DAF, and 195 DAF were selected for metabolic and transcriptomic analyses to explore the dynamic changes in FLL active components and the functions of key genes. A quantitative analysis of the main compounds was performed using high performance liquid chromatography (HPLC). The compounds and genes related to the biosynthesis of secoiridoids, flavonoids and triterpenoids were analyzed by combining metabolites with the transcriptome. Based on the GTs of *Rhodiola rosea*, we identified nine homologs in FLL and showed that these GTs catalyze the formation of salidroside and hydroxysalidroside from tyrosol and hydroxytyrosol, respectively. The results revealed that changes in their compound contents and in the expression of FLL-related genes complied with certain rules across the different fruit development periods These results provide a basis for the efficient utilization of FLL across different seasonal collection times and diversified development and utilization of FLL.

## Results

### Metabolite accumulation during FLL development

The metabolites present in six fruit development periods of FLL were analyzed *via* LC-MS/MS, and the mixture of the extracts from each sampling period was used as a quality control sample to identify the compounds. The total ion chromatogram of the quality control mixture in the negative and positive modes is exhibited in Fig. S2. Based on the standards and previous researches, a total of 70 compounds were identified, of which secoiridoids (40%), flavonoids (14%), and phenylethanoids (11%) were the most abundant. Details of the identified metabolites are listed in Table 1. The MS/MS spectra of compounds identified using the standards are shown in Fig. S3. The PCA analysis showed that 36 samples (six fruit development periods with six replicates) were separated. The samples in each period were grouped into one cluster, showing a trend of separation among metabolic groups (S4A).

**Fig. 1:**
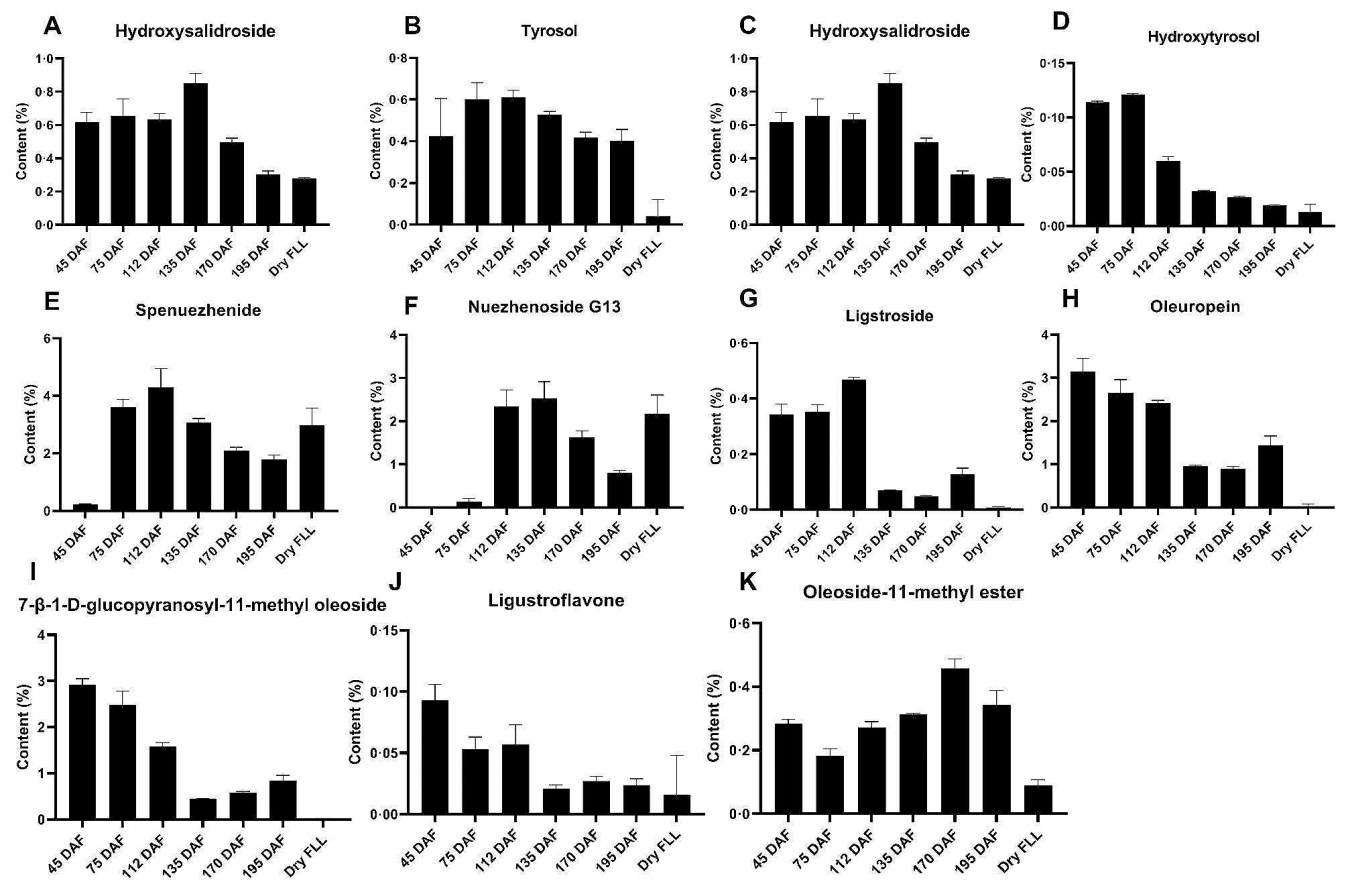
The content variation of the main compounds (*n* = 6). The x-axis shows the six fruit development periods of FLL, with dry FLL representing the traditional Chinese medicine (TCM) of FLL; the y-axis shows the mass percentage of the compound content. Data are represented as mean values with standard deviation obtained from six biological replicates. FLL, Fructus Ligustri Lucidi; DAF, days after flowering

To explore the variations in the main compounds across the development periods of FLL, tyrosol, salidroside, hydroxysalidroside, hydroxytyrosol, specnuezhenide, oleuropein, nuezhenoside G13, ligstroside, ligustroflavone, oleoside-11-methyl ester, and 7-*β*-1-D-glucopyranosyl-11-methyl oleoside were quantified using HPLC based on a previous method [9], with dry FLL being used as a control. Spenuezhenide was the standard of the Chinese Pharmacopoeia (2020), and the abundance of this compound was 4.3% at 112 DAF. Across sampling periods, spenuezhenide accumulated rapidly at 75 DAF (Fig. 1), reaching the highest level at 112 DAF, and then decreasing gradually. Oleuropein accumulated at 45 DAF, and then decreased gradually, after which it increased at 195 DAF. Nuezhenoside G13 accumulated rapidly at 112 DAF, reached its highest level at 135 DAF (2.5343%), then decreased gradually. The dry FLL had a high content of nuezhenoside G13 and specnuezhenide. The oleuropein content in dry FLL was extremely low (0.0272%), while its abundance in fresh FLL was significant (0.8945–3.1503%). Tyrosol content increased from 45 DAF to 112 DAF, then decreased from 112 DAF to 195 DAF. Salidroside and hydroxytyrosol accumulated mainly at 45 DAF and 75 DAF, then rapidly decreased at 112 DAF, remaining at stable levels from 112 DAF to 195 DAF. The hydroxysalidroside content increased from 45 DAF to 135 DAF, then decreased gradually from 135 DAF to 195 DAF. The tyrosol, salidroside, hydroxytyrosol, and hydroxysalidroside contents in dry FLL were lower than those at 45 DAF and 75 DAF. Oleoside-11-methyl ester and 7-*β*-1-D-glucopyranosyl-11-methyl oleoside (the glycosylated product of oleoside-11-methyl ester) are known to be involved in the biosynthesis of oleoside-type secoiridoids [10, 11]. The content of 7-*β*-1-D-glucopyranosyl-11-methyl oleoside decreased from 45 DAF to 135 DAF, then slightly increased to 195 DAF; it was the lowest in dry FLL. The oleoside-11-methyl ester content decreased from 45 DAF to 75 DAF, reached its highest level at 170 DAF, then decreased again at 195 DAF; its content in dry FLL was lower than that in the six periods. The trends in the LC-MS-based intensities of the compounds mentioned above, across development stages, were similar to their variation in content (Fig. S4B).

**Table 1:** Details of the identified metabolites of FLL

Rt (min)	Formula	Calculated mass (m/z)	Error (ppm)	Mode	Identification	Observed fragment ions in MS and MS/MS (m/z)	Class
5.06	C_10_H_14_O_5_	214.0841	0.9	[M-H]^−^	Nuzhenal A	139.0772, 111.0821, 107.0864	Iridoids
5.62	C_16_H_26_O_8_	346.1628	1.3	[M + H]^+^	Oleuropeic acid 8-*O*-glucoside	347.1675	Iridoids
5.69	C_14_H_20_O_8_	316.1158	-0.7	[M-H]^−^	Hydroxysalidroside^a^	59.0150 135.0454, 89. 0241, 214.9202	Phenylethanoids
5.87	C_14_H_22_O_7_	302.1366	1.2	[M + H]^+^	Liguluciridoid B	303.1408	Secoiridoids
5.92	C_17_H_24_O_14_	452.1166	5.4	[M-H]^−^	Nuezhenidic acid	153.0551,123.0446,151.0393	Secoiridoids
6.13	C_11_H_14_O_4_	210.0892	0.5	[M + H]^+^	Sinapyl alcohol	79.0573, 107.0718,105.0719	Volatile oils
6.14	C_11_H_16_O_5_	228.0998	1.7	[M + H]^+^	Nuzhenal C	109.0661, 169.0860, 155.0711, 139.0764	Iridoids
6.31	C_8_H_10_O_3_	154.0630	-1.1	[M-H]^−^	Hydroxytyrosol^a^	123.0445	Phenylethanoids
6.32	C_16_H_24_O_10_	376.1370	0.3	[M-H^]−^	Loganic acid/7-*epi*-Loganic acid^a^	213.0768, 375.1293, 69.0358, 169.0871	Iridoids
6.52	C_14_H_20_O_7_	300.1209	-2.3	[M-H]^−^	Salidroside^a^	59.0161, 119.0498, 89.0246	Phenylethanoids
6.56	C_16_H_22_O_10_	374.1213	0.9	[M + HCOO]^−^	7-Ketologanic acid	59.0159, 89.0254	Iridoids
6.57	C_27_H_30_O_17_	626.1483	0.6	[M-H]^−^	Quercetin-3-*O*-sophoroside	625.1430, 299.0203, 462.0822	Flavonoids
6.96	C_18_H_26_O_11_	418.1475	0.9	[M-H]^−^	Oleoside dimethyl ester	209.0811, 417.1478, 161.0256	Secoiridoids
7.02	C_17_H_24_O_10_	388.1370	-0.6	[M + H]^+^	Ketologanin	165.0544, 151.0387	Iridoids
7.03	C_18_H_28_O_9_	388.1733	2.9	[M + H]^+^	Nuezhengalaside	389.1783, 165.0534	Secoiridoids
7.07	C_23_H_34_0_16_	566.1847	0.7	[M-H]^−^	7-*β*-1-D-Glucopyranosyl-11-methyl oleoside^a^	403.1247, 179.0561, 223.0614, 89.0253	Secoiridoids
7.1	C_11_H_14_O_6_	242.0790	0.6	[M + H]^+^	Elenolic acid	151.0393, 165.0558	Secoiridoids
7.13	C_16_H_22_O_11_	390.1162	1.4	[M-H]^−^	Oleoside	59.0154, 69.0353, 121.0643	Secoiridoids
7.37	C_17_H_24_O_12_	420.1268	-0.6	[M + HCOO]^−^	10-Hydroxyoleoside 11-methyl ester	209.0458, 241.0724, 177.0198	Secoiridoids
7.48	C_9_H_8_O_3_	164.0473	1.6	[M-H]^−^	*p*-Coumaric acid	119.0489, 93.0344, 145.8909	Phenylpropanoids
7.61	C_8_H_8_O_4_	168.0423	-1.1	[M + H]^+^	Vanillic acid	111.0086	Benzoic acids
7.76	C_11_H_14_O_6_	242.0790	0.6	[M-H]^−^	Eleanolic acid	68.9995, 101.0245, 139.0397, 209.0421	secoiridoids
7.8	C_17_H_26_O_10_	390.1526	-0.5	[M + HCOO]^−^	7-*epi*-Loganin/loganin^a^	227.0924, 130.9653, 304.9140	Iridoids
7.96	C_35_H_46_O_20_	786.2582	2.4	[M-H]^−^	Echinacoside	785.2527, 623.2189, 787.0911	Phenylethanoids
8.18	C_17_H_24_O_11_	404.1319	5.2	[M-H]^−^	Oleoside-11-methyl ester^a^	223.0609, 179.058, 59.0171	Secoiridoids
8.27	C_10_H_12_O_3_	180.0786	-3.0	[M + H]^+^	Tyrosyl acetate	67.0593, 91.0570, 93.0741	Phenylethanoids
8.31	C_15_H_12_O_7_	304.0583	-0.9	[M + H]^+^	Taxifolin	153.0181, 123.0460	Flavonoids
8.32	C_10_H_14_O_4_	198.0892	-0.1	[M-H]^−^	*δ*-Valerolactone	153.0925, 197.0811	Secoiridoids
8.63	C_17_H_24_O_11_	404.1319	-2.9	[M-H]^−^	Secoxyloganin^a^	121.0281, 371.0977, 165.0554, 59.0155	Secoiridoids
9.14	C_33_H_40_O_19_	740.2164	0.1	[M + H]^+^	Mauritianin	741.2201, 579.1687	Flavonoids
9.21	C_27_H_30_O_16_	610.1534	-2.6	[M-H]^−^	Rutin	300.0268, 609.1464	Flavonoids
9.62	C_31_H_42_O_18_	702.2371	0.8	[M-H]^−^	Neonuezhenide	315.1069	Secoiridoids
9.98	C_29_H_36_O_15_	624.2054	0.2	[M-H]^−^	Verbascoside/Isoverbascoside	161.0235, 623.1947, 461.1645	Phenylethanoids
10.12	C_21_H_20_O_11_	448.1006	6.0	[M-H]^−^	Luteolin 7-glucoside	285.0410, 447.0920	Flavonoids
10.15	C_33_H_40_O_18_	724.2215	1.0	[M-H]^−^	Ligustroflavone^a^	723.2202, 269.0455	Flavonoids
10.18	C_18_H_20_O_8_	364.1158	0.0	[M-H]^−^	Demethyl-oleuropeindial	151.0391, 183.0662	Secoiridoids
10.23	C_25_H_30_O_15_	570.1585	1.1	[M-H]^−^	Oleuropeinic acid	151.0390, 177.0182, 165.0549	Secoiridoids
10.45	C_33_H_44_O_18_	728.2528	8.0	[M-H]^−^	6’’’-Acetylnicotiflorine	681.1236, 223.0626, 681.1023	Secoiridoids
10.47	C_25_H_32_O_14_	556.1792	-0.7	[M-H^]−^	10-Hydroxyoleuropein	273.0755, 89.0246, 181.0499	Secoiridoids
10.53	C_9_H_8_O_4_	180.0423	2.2	[M + H^]+^	Caffeic acid	121.0290, 53.0056	Phenylethanoids
10.57	C_24_H_30_O_13_	526.1686	-1.8	[M + HCOO]^−^	Demethyloleuropein	179.0350, 151.0400, 135.0450, 153.0194, 571.1656	Secoiridoids
10.59	C_27_H_30_O_15_	594.1585	0.6	[M-H]^−^	Luteolin-7-*O*-rutinoside	593.1505, 555.1727, 413.0861	Flavonoids
10.72	C_27_H_30_O_14_	578.1636	-4.7	[M + H]^+^	Apigenin-7-*O*-rutinoside	417.1146, 579.1682	Flavonoids
11.11	C_31_H_42_O_17_	686.2422	0.1	[M-H]^−^	Specnuezhenide^a^	453.1406, 685.2410, 523.4183	Secoiridoids
12.12	C_25_H_30_O_14_	554.1636	-3.6	[M-H]^−^	Ligustrosidic acid/Isoligustrosidic acid	165.0561, 537.1978, 150.0330	Secoiridoids
12.15	C_21_H_20_O_10_	432.1057	-0.1	[M-H]^−^	Apigenin 7-glucoside	268.0382	Flavonoids
12.36	C_17_H_20_O_6_	320.1260	0.7	[M + HCOO]^−^	Oleacein	135.0455, 137.0616, 211.0615, 179.0357	Secoiridoids
14.56	C_25_H_32_O_13_	540.1843	-2.2	[M-H]^−^	Oleuropein^a^	539.1809, 307.0839, 377.1261, 149.0250	Secoiridoids
14.56	C_19_H_22_O_8_	378.1315	-1.0	[M-H]^−^	Oleoeuropein aglycone	139.0039, 149.0244, 275.0580	Secoiridoids
16.42	C_19_H_22_O_8_	378.1315	-0.9	[M-H]^−^	Oleuropeindial	95.0511, 111.0090, 139.0037, 149.0254, 101.0232	Secoiridoids
16.67	C_48_H_64_O_27_	1072.3635	5.4	[M-H]^−^	Nuezhenoside G13^a^	1071.3633, 685.2377, 523.1831	Secoiridoids
17.37	C_25_H_32_O_12_	524.1894	3.5	[M-H]^−^	Ligstroside^a^	291.0882, 101.0247, 259.0975	Secoiridoids
17.51	C_15_H_10_O_6_	286.0477	-2.0	[M-H]^−^	Kaempferol	285.0395, 133.0289, 151.0032	Flavonoids
17.55	C_19_H_22_O_7_	362.1366	-7.6	[M-H]^−^	Oleokoronal/Ligstroside aglycon	291.0882, 101.0247	Secoiridoids
20.85	C_22_H_24_O_8_	416.1471	0.5	[M + H]^+^	8-Acetoxypinoresinol	417.1496, 385.1235	Lignans
21.05	C_42_H_54_O_22_	910.3107	0.4	[M-H]^−^	6’-Elenolylnicotiflorine	909.3027, 291.0880, 361.1292	Secoiridoids
25.02	C_13_H_18_O	190.1358	0.6	[M-H]^−^	Damascenone	132.0577, 189.1261	Volatile oils
25.14	C_13_H_20_O_3_	224.1412	-2.4	[M + H]^+^	Vomifoliol	225.1466, 209.0256, 195.0994	Volatile oils
25.73	C_30_H_48_O_5_	488.3502	0.2	[M-H]^−^	Tormentic acid	487.3438, 469.3326, 423.3281	Triterpenoids
26.13	C_12_H_20_O_2_	196.1463	-0.7	[M-H]^−^	Linalyl acetate/L-Bornyl acetate	195.1404, 130.9930, 138.0692	Volatile oils
27.74	C_30_H_48_O_4_	472.3553	2.1	[M + H]^+^	Pomolic acid/2*α*-Hydroxyoleanolic acid	201.1633, 455.3497, 187.1479, 409.3454	Triterpenoids
29.91	C_16_H_22_O_4_	278.1518	0.5	[M-H]^−^	Dibutyl phthalate	149.0237	Volatile oils
29.98	C_31_H_50_O_4_	486.3709	0.7	[M-H]^−^	*α*-Ursolic acid methyl ester	453.3384, 451.3222, 485.3663	Triterpenoids
31.51	C^32^H^50^O^5^	514.3658	0.5	[M + H]^+^	19-Hydroxy-3-acetylursolic acid	455.3495, 201.1617, 515.3280, 409.3425	Triterpenoids
33.45	C_30_H_48_O_3_	456.3604	-1.6	[M + HCOO]^−^	Betulinic acid	455.3519	Triterpenoids
33.54	C_30_H_48_O_3_	456.3604	0.7	[M-H]^−^	Oleanolic acid/Ursolic Acid^a^	455.3540	Triterpenoids
33.59	C_15_H_24_	204.1878	-0.9	[M + H]^+^	*cis-*Thujopsene/*α*-Humulene	149.0245, 121.0284	Volatile oils
37.98	C_32_H_50_O_4_	498.3709	-0.2	[M-H]^−^	3-Acetylursolic acid/Acetyloleanolic acid	497.366	Triterpenoids
38.13	C_30_H_50_O	426.3862	-0.9	[M + H]^+^	*β*-Amyrin/*α*-Amyrin/Lupeol	427.3926	Triterpenoids
38.94	C_18_H_34_O_2_	282.2559	2.7	[M-H]^−^	Oleic acid	281.2481	Fatty acids

^a^ denotes compounds that were compared with the standard substance; Rt represents retention time

### Transcriptome analysis and annotations of different FLL development periods

To explore the underlying mechanisms for the changes in the levels of the major compounds at the gene level, transcriptome analysis of six FLL development periods was performed. The analysis generated 112.3 Gb of clean data, and the Q30 base percentage was more than 93% (Table S3). A total of 103,058 unigenes were assembled, with the length of the unigenes ranging from 301 to 16,743 bp. All unigenes were annotated using common databases, including the NR (NCBI non-redundant protein sequences), NT (NCBI nucleotide sequences), KO (euKaryotic Ortholog groups), SwissProt, PFAM, GO (Gene Ontology), and KEGG (Kyoto Encyclopedia of Genes and Genomes), to which approximately 48, 45.45, 15.36, 32.52, 32.12, 32.12, and 8.24 of the unigenes were mapped, respectively (Table S4). Inter-sample correlation analysis of the 18 samples showed good biological duplication within groups (Fig. S5).

Differentially expressed genes (DEGs) were selected using the parameters |log2Fold Change| ≥ 1 and FDR < 0.05, with 36,540 unigenes were screened (Table S5). There were 25,783 unigenes in NR, 24,791 in NT, 8,301 in KO, 5,351 in KEGG, 18,865 in SwissProt, 18,009 in PFAM, and 13,371 in GO that were annotated among the DEGs. The number of DEGs were 5,854, 5,467, 1,214, 6,143, and 1,687 between 45 DAF and 75 DAF, 75 DAF and 112 DAF, 112 DAF and 135 DAF, 135 DAF and 170 DAF, and 170 DAF and 195 DAF, respectively. A total of 198 KEGG pathways were mapped, with the most enriched pathways being related to glucose metabolism, fatty acid biosynthesis, and terpenoid biosynthesis (Table S6).

### Analysis of the triterpenoid biosynthesis pathway

**Fig. 2:**
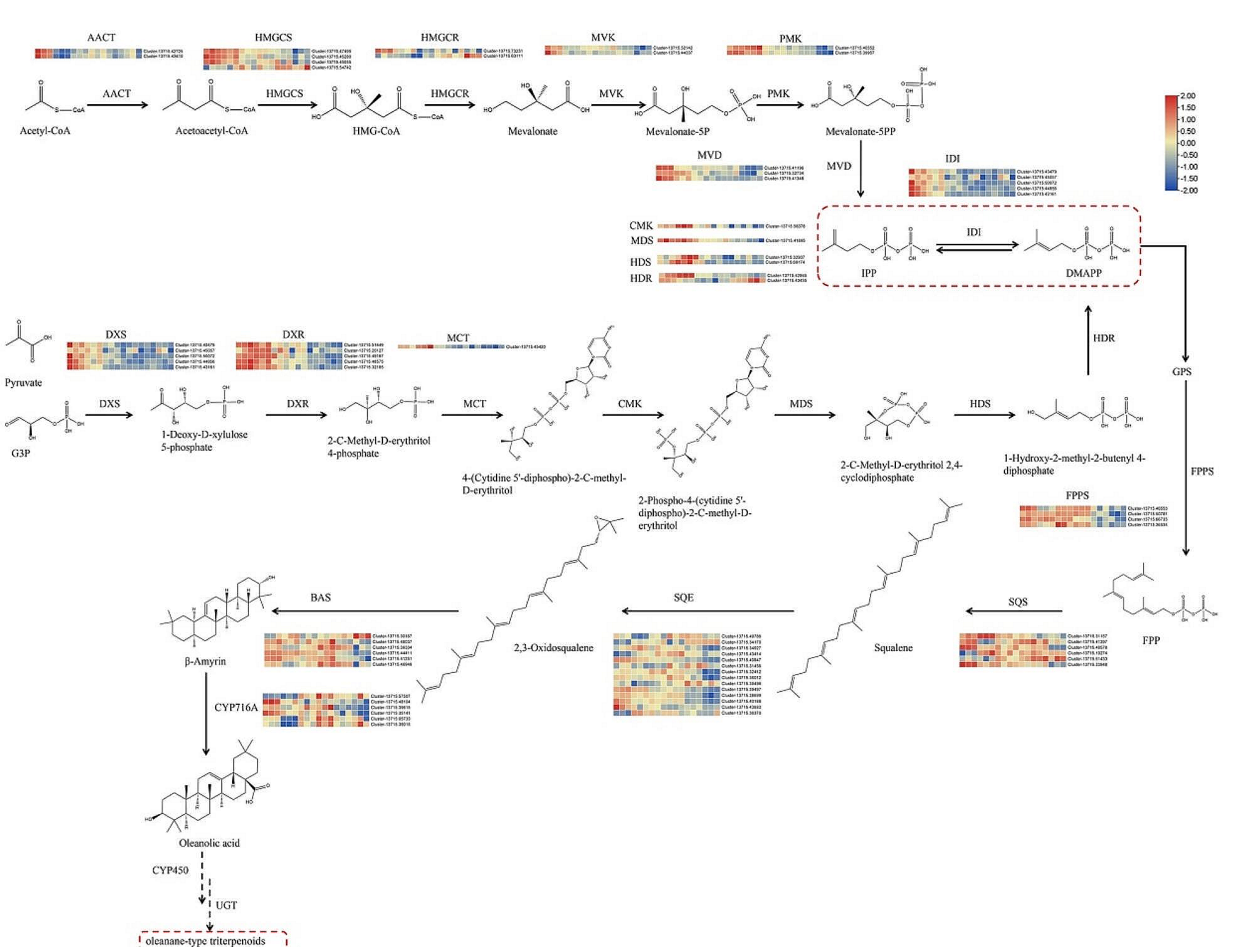
The biosynthesis of oleanolic acid and its related DEG expression during six stages. Highly expressed genes are indicated in red, while genes with reduced expression. The color scales reflect a log_2_-transformed mean of fragments per kilobase of transcript per million mapped (FPKM) values. From left to right, the sample sequence is 45 DAF_1, 45 DAF_2, 45 DAF_3, 75 DAF_1, 75 DAF_2, 75 DAF_3, 112 DAF_1, 112 DAF_2, 112 DAF_3, 135 DAF_1, 135 DAF_2, 135 DAF_3, 170 DAF_1, 170 DAF_2, 170 DAF_3, 195 DAF_1, 195 DAF_2 and 195 DAF_3. DAF, days after flowering

Triterpenoids, such as oleanolic acid, are important secondary metabolites of FLL. Triterpenoids are generated from 2,3-oxidosqualene, which is mainly derived from the cytosolic mevalonic acid (MVA) pathway and, to a smaller extent, the plastidial methylerythritol phosphate (MEP) pathway. In the MVA pathway, the conversions of isopentenyl diphosphate (IPP) and dimethylallyl diphosphate (DMAPP) are catalyzed by acelyl-CoA *C*-acetyltransferase (AACT), hydroxymethylglutaryl-CoA synthase (HMGCS), hydroxymethylglutaryl-CoA reductase (HMGCR), mevalonate kinase (MVK), and phosphomevalonate kinase (PMK) to generate sesquiterpenes and triterpenes. In the MEP pathway, IPP and DMAPP conversions are catalyzed by 1-deoxy-D-xylulose-5-phosphate synthase (DXS), 1-deoxy-D-xylulose-5-phosphate reductase (DXR), 2-*C*-methyl-D-erythritol 4-phosphate cytidylyltransferase (MCT), 4-diphosphocytidyl-2-*C*-methyl-D-erythritol kinase (CMK), 2-*C*-methyl-D-erythritol 2,4-cyclodiphosphate synthase (MDS), (E)-4-hydroxy-3-methylbut-2-enyl-diphosphate synthase (HDS), and 4-hydroxy-3-methylbut-2-en-1-yl diphosphate reductase (HDR). IPP and DMAPP are then catalyzed to FPS by farnesyl pyrophosphate synthase (FPPS). Finally, 2,3-oxidosqualene can be obtained using squalene monooxygenase (SQE) and squalene synthase (SQS) catalysis (Fig. 2). The precursor produced can then be consumed by beta-amyrin synthase (BAS) and CYP716A to produce oleanolic acid. The expression levels of these genes (Fig. 2) showed that the genes related to the MEP and the MVA pathways were highly expressed at 45 DAF, 75 DAF, and 112 DAF, when the color of FLL was still green. The expression of downstream genes like *FPPS*, *SQS*, *SQE*, *BAS*, and *CYP716A* increased as fruit developed, suggesting that metabolites were gradually catalyzed in the direction of oleanolic acid. The transcript Cluster-13715.33948, Cluster-13715.40847, Cluster-13715.44411, and Cluster-13715.48104, annotated as *SQS*, *SQE*, *BAS*, and *CYP716A*, respectively, were analyzed using qRT-PCR, and their trends of gene expression trends were consistent with the RNA-seq data (Fig. S6).

### Analysis of the secoiridoid biosynthesis pathway

**Fig. 3:**
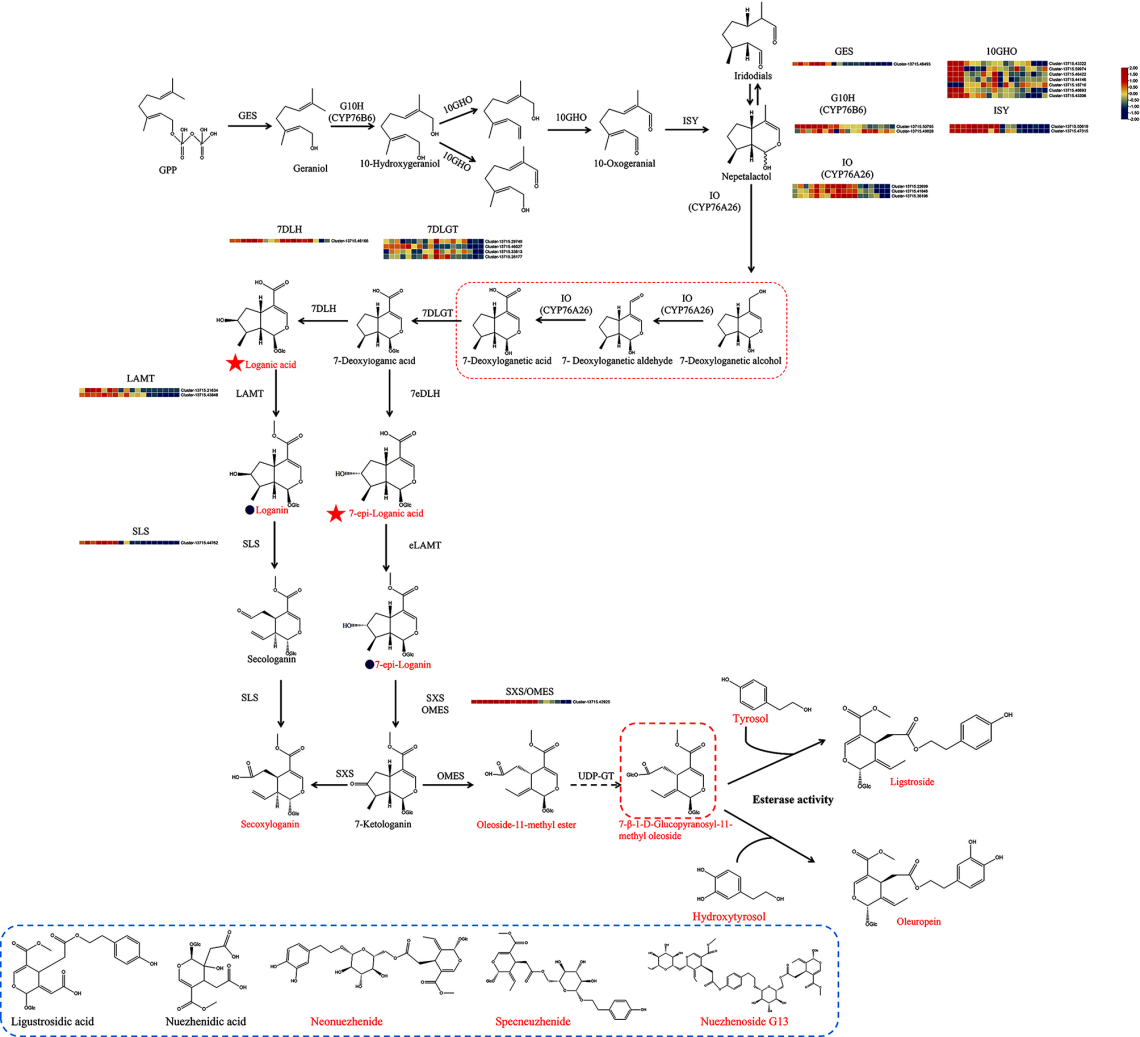
Biosynthesis of secoiridoids and expression of related DEGs across the six periods of FLL. Compounds in red were detected in FLL. Red five-pointed stars indicate that the compounds were isomers and could not be distinguished using LC-MS. The words in red indicate compounds that were identified using the standards. The pentagram and the circle-marked compounds indicate that they are isomers. Highly expressed genes are indicated in red, while genes with reduced expression are in blue. The color scales reflect a log_2_-transformed mean of fragments per kilobase of transcript per million mapped (FPKM) values. From left to right, the sample sequence is 45 DAF_1, 45 DAF_2, 45 DAF_3, 75 DAF_1, 75 DAF_2, 75 DAF_3, 112 DAF_1, 112 DAF_2, 112 DAF_3, 135 DAF_1, 135 DAF_2, 135 DAF_3, 170 DAF_1, 170 DAF_2, 170 DAF_3, 195 DAF_1, 195 DAF_2, and 195 DAF_3. FLL, Fructus Ligustri Lucidi; DAF, days after flowering

Secologanin accumulates mainly in *Catharanthus roseus*, and its biosynthetic pathway has been elucidated [12]. The structure of oleoside-type secoiridoids is similar to secologanin-type secoiridoids [11]. The early biosynthetic steps for these two compounds are the same, and they are both converted from geraniol, which is catalyzed by geraniol synthase (GES) to produce geranyl diphosphate (GPP). Geraniol then reacts with geraniol 10-hydroxylase (G10H), 10-hydroxygeraniol oxidoreductase (10GHO), and iridoid synthase (ISY) to produce nepetalactol [13]. The biosynthesis of secologanin from nepetalactol has been reported. Nepetalactol is catalyzed by iridoid oxidase (IO), 7-deoxyloganetic acid glucosyltransferase (7DLGT), 7-deoxyloganic acid 7-hydroxylase (7DLH), loganic acid *O*-methyltransferase (LAMT), and secologanin synthase (SLS) to generate secologanin [14]. Portions of the pathways downstream of oleoside-type secoiridoid biosynthesis have been proposed in *Olea europaea* L. [11], starting from 7-deoxyloganic acid which is catalyzed by 7-deoxy-loganic acid 7-*epi*-hydroxylase (7eDLH), 7-*epi*-loganic acid methyl-transferase (eLAMT), oleoside methyl ester synthase (OMES), and secoxyloganin synthase (SXS) to produce oleoside-11-methyl ester (OME) (Fig. 3). Then, according to changes in the molecular structure, oleuropein or ligstroside may be synthesized through glycosylation and esterification processes. In our study, *GES, G10H, 10GHO, ISY*, and *IO* tended to be expressed in earlier days after flowering in FLL (45 DAF, 75 DAF, and 112 DAF) and were down-regulated in later days after flowering (135 DAF, 170 DAF, and 195 DAF). Based on the Blast results of Cr7DLH (AGX93062.1), CrLAMT (AGX93063.1), CrSLS (AAA33106.1), Oe7eDLH (ALV83449.1), OeOMES (QOW17548.1), OeSXS (QOW17550.1) and OeLAMT (AFS28695.1), the Oe7eDLH and Cr7DLH homologs in FLL were the same (Cluster-13715.46166) and were highly expressed at 135 DAF and 170 DAF. The homologous genes of OeLAMT and CrLAMT were also the same (Cluster-13715.21634 and Cluster-13715.43848), being highly expressed from 45 DAF to 112 DAF. OeOMES and OeSXS were aligned to the same gene, Cluster-13715.42925, and denoted as OMES/SXS. The expression levels of *CrSLS* homologous (Cluster-13715.44762) and OMES/SXS were similar to those of ISY (Cluster-13715.50819 and Cluster-13715.47315). The compounds catalyzed by the enzymes above, such as loganic acid, loganin, secoxyloganin, and oleoside-11-methyl ester were identified *via* LC-MS using standards. However, as loganic acid, loganin, secologanin, and secoxyloganin were the isomers of 7-*epi*-loganic acid, 7-*epi*-Loganin, 7-ketologanin, and oleoside-11-methyl ester, respectively. They were not divided by the standards *via* LC-MS (except for secoxyloganin and oleoside-11-methyl ester). Combined with the gene expression and compound contents analysis, the content of compounds such as oleuropein, ligstroside, neonuezhenide, and specneuzhenide exhibited a positive correlation with several genes including GES, G10H, IO, ISY, 7DLGT, LAMT, SLS, and SXS/OMES (Fig. S7 and Table S7). In addition, the Pearson’s correlation coefficient (r) between *SLS* and *ISY* (Cluster-13715.50819) was 0.89, and between *SLS* and *ISY* (Cluster-13715.47315) was 0.97; r between *SXS/OMES* and *ISY* (Cluster-13715.50819) was 0.82, between *SXS/OMES* and *ISY* (Cluster-13715.47315) was 0.86, suggesting that these genes may be co-expressed. In order to validate the gene expression in transcriptome, *GES* (Cluster-13715.46493), *10GHO* (Cluster-13715.43322), and *IO* (Cluster-13715.22699) were performed using qRT-PCR (Fig. S6) with a similar expression trend as in the transcriptome.

### Analysis of the phenylethanol biosynthesis pathway

**Fig. 4:**
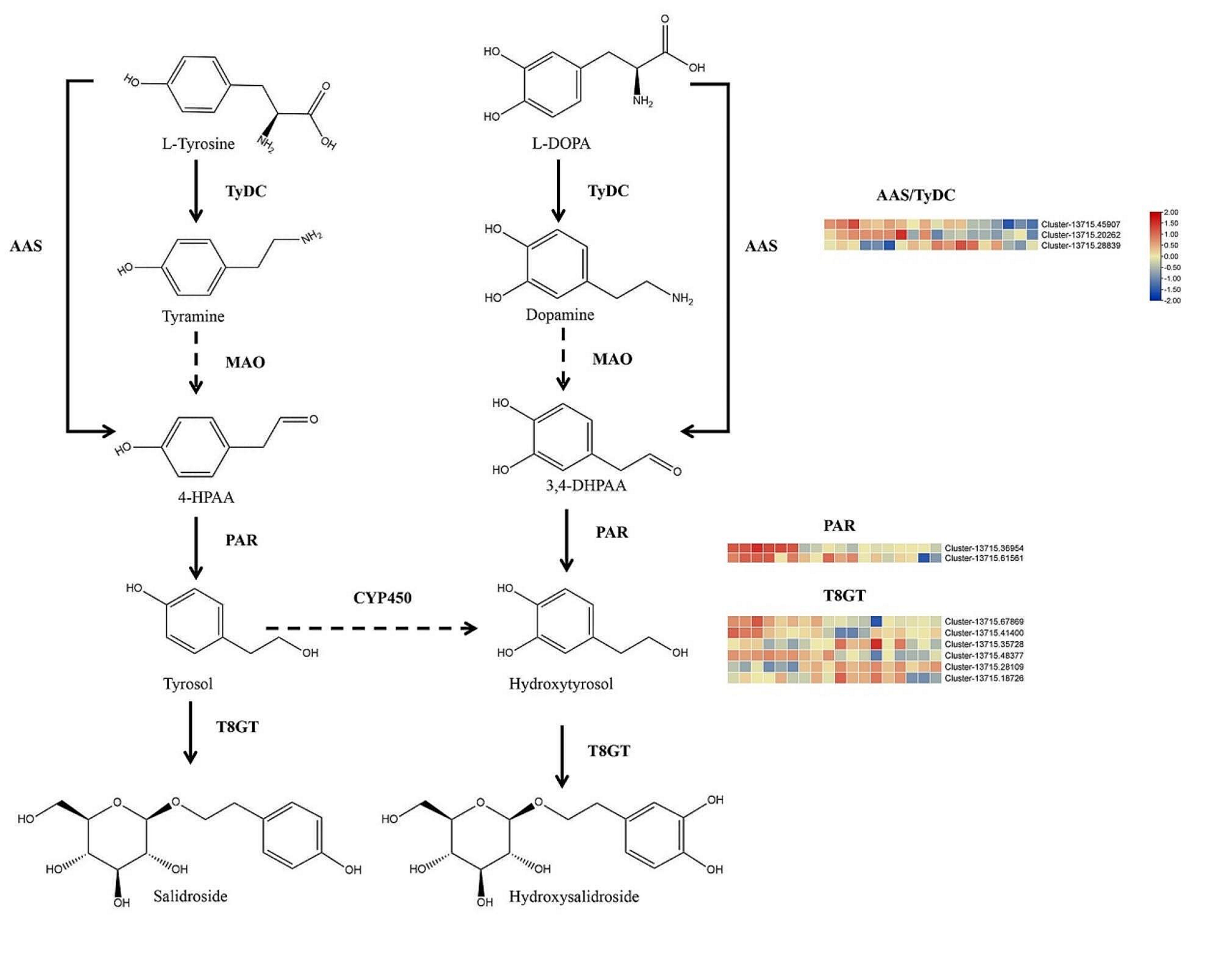
The biosynthesis of salidroside and hydroxylsalidroside. Highly expressed genes are indicated in red, while genes with reduced expression are in blue. The color scales reflect a log_2_-transformed mean of fragments per kilobase of transcript per million mapped (FPKM) values. From left to right, the sample sequence is 45 DAF_1, 45 DAF_2, 45 DAF_3, 75 DAF_1, 75 DAF_2, 75 DAF_3, 112 DAF_1, 112 DAF_2, 112 DAF_3, 135 DAF_1, 135 DAF_2, 135 DAF_3, 170 DAF_1, 170 DAF_2, 170 DAF_3, 195 DAF_1, 195 DAF_2, and 195 DAF_3. DAF, days after flowering

Phenylethanols and glycosides are classes of bioactive components in FLL that typically contain a substructure of tyrosol, hydroxyltyrosol, salidroside, and hydroxylsalidroside. The biosynthesis of hydroxysalidroside is similar to that of salidroside. Tyrosine/Dopa decarboxylase (TyDC) can catalyze the conversions of l-tyrosine and l-3,4-dihydroxyphenylalanine to tyramine and dopamine, respectively. Then, 4-hydroxyphenylacetaldehyde (4-HPAA) and 3,4 dihidroxyphenylacetaldehyde (3,4-DHPAA) can be generated from tyramine and dopamine by monoamino oxidase (MAO). Both 4-HPAA and 3,4-DHPAA are catalyzed by phenylacetaldehyde reductase (PAR) to generate tyrosol and hydroxytyrosol. The structure of hydroxyltyrosol contains an extra C3-OH as compared to tyrosol. Glycosylation of the above two compounds (tyrosol and hydroxyltyrosol) at the C8 position by T8GT (UDP-glucose 8-*O*-glucosyltransferase) generates salidroside and hydroxylsalidroside (Fig. 4, Fig. S8A-B). T4GT (UDP-glucose 4-*O*-glucosyltransferase) was identified in *R. rosea* as converting tyrosol to icariside D, a structural isomer of salidroside [15]. Recently, aromatic aldehyde synthase (AAS) has been shown to directly convert l-tyrosine and l-DOPA to 4-HPAA and 3,4-DHPAA, respectively [16]. The compounds related to biosynthesis mentioned above, such as tyrosol, hydroxyltyrosol, salidroside, and hydroxylsalidroside were identified *via* LC-MS and HPLC (Fig. S9 and Table 1).

TyDC and AAS belong to the AAAD family of proteins. Owing to the high amino acid sequence identity between individual AAAD paralogs, the annotation of TyDC and AAS can be confusing. Genes such as Cluster-13715.45907, Cluster-13715.20262, and Cluster-13715.28839 were identified as *TyDC/AAS*. The *MAO* was not annotated or blasted in our RNA-seq data, while *PAR* expression was also found to be similar to *TyDC/AAS* expression. Based on T4GT and T8GT sequences in *R. rosea*, Cluster-13715.67869, Cluster-13715.41400, Cluster-13715.48377, Cluster-13715.35728, Cluster-13715.18726, and Cluster-13715.28109 were selected to being T8GT-like, and Cluster-13715.43413, Cluster-13715.51696, Cluster-13715.7146 were denoted to be T4GT-like. Cluster-13715.67869, Cluster-13715.41400, and Cluster-13715.48377 were highly expressed in 45 DAF, which then gradually decreased in the later months. In contrast, Cluster-13715.35728, Cluster-13715.18726, and Cluster-13715.28109 were highly expressed in 135 DAF, 170 DAF, and 195 DAF (Fig. 4). Cluster-13715.43413 and Cluster-13715.51696 were highly expressed in 45 DAF, and then their expression gradually decreased. Cluster-13715.7146 was highly expressed at 170 DAF (Fig. S8C). It seems that the variation in the contents of salidroside and hydroxylsalidroside may be caused by the differential expression of these GTs over the six months.

### Analysis of flavonoid biosynthesis

Flavonoids, which are common chemical constituents of FLL, have also been isolated and studied; these including kaempferol, ligustroflavone, luteolin 7-glucoside [17]. Ten flavonoids were detected in FLL across the different periods. In the transcriptome analysis, KEGG pathways related to flavonoids were related to flavonoid biosynthesis (ko00941) (Fig. S10). DEGs related to the above pathways are marked in red (Fig. S10). The genes encoding proteins related to anthocyanin generation, such as flavanone 3-hydroxylase (F3H), flavonoid 3′-hydroxylase (F3′H), dihydroflavonol 4-reductase (DFR), anthocyanidin synthase (ANS), and UDP-glucose: flavonoid 3-*O*-glucosyltransferase (UFGT), were highly expressed from 135 DAF to 195 DAF. The expression of these genes was consistent with the color change in FLL, wherein the color of the exocarp changed from lavender (135 DAF) to black-purple (195 DAF) (Fig. S11, Fig. S1D-F).

### Glucosyltransferase selection and characterization

Secoiridoids, phenylethanoids, and their C8-glycosides were enriched in FLL. Tyrosol, hydroxyltyrosol, their glycosylated production salidroside and hydroxylsalidroside, also participate in the biosynthesis of secoiridoids to produce oleuropein, ligstroside, specneuzhenide, and neonuezhenide. Therefore, GTs play an important role in the biosynthesis of secoiridoids and phenylethanoids. In exploring the homologous genes of RrT8GT (AUI41147.1) and RrT4GT (AUI41117.1), it was found that Cluster-13715.67869, Cluster-13715.41400, Cluster-13715.48377, Cluster-13715.35728, Cluster-13715.18726, and Cluster-13715.28109 were similar to RrT8GT (noted as T8GTs); while Cluster-13715.43413, Cluster-13715.51696, and Cluster-13715.7146 were similar to RrT4GT (noted as T4GTs) (Fig. S8C). In theory, tyrosol and hydroxyltyrosol can be glycosylated at C4 to produce icariside D2 and hydroxytyrosol 4-*O*-glucoside, respectively, although they were not detected in FLL.

**Fig. 5:**
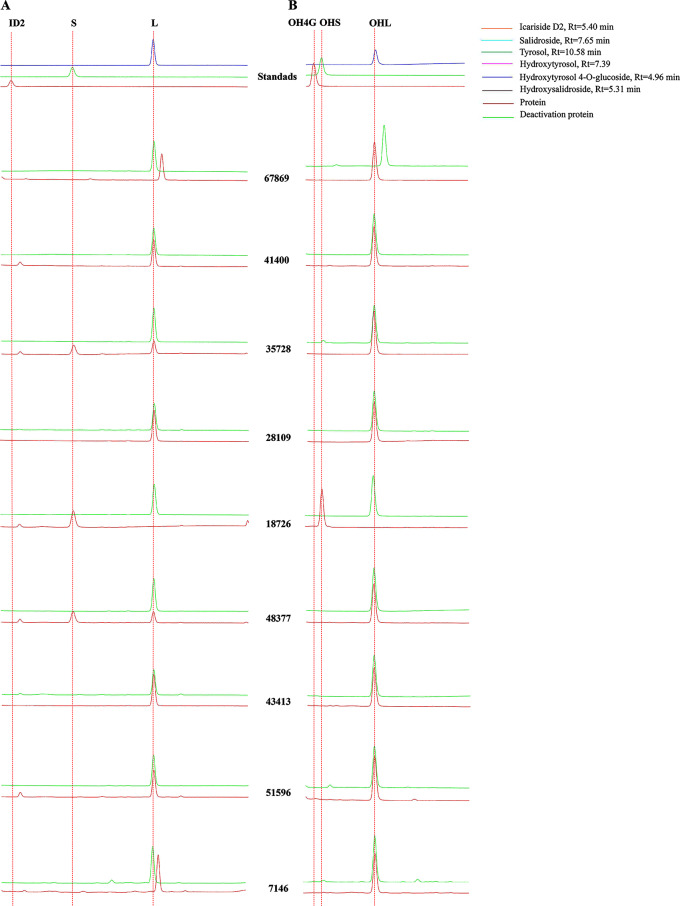
HPLC analysis of the reaction products of the T8GTs and T4GTs. ID2, icariside D2; S, salidroside; L, tyrosol; OH4G, hydroxytyrosol 4-*O*-glucoside; OHS, hydroxysalidroside; OHL, hydroxyltyrosol. Rt represents the retention time

We examined the biochemical activity of the above T8GTs and T4GTs above with recombinant enzymes expressed in *E. coli*, using tyrosol or hydroxyltyrosol as substrates. HPLC analyses of the enzymatic activity assay revealed that the recombinant enzyme 67,869 could completely metabolize tyrosol and hydroxyltyrosol completely to unknown compounds 1 and 2, respectively, with an Rt of 10.82 min and 7.79 min, respectively; 18,726 reacted with tyrosol and hydroxyltyrosol completely to produce salidroside and hydroxylsalidroside; 48,377 catalyzde most of the tyrosol to salidroside, but could not catalyze hydroxytyrosol; 41,400 and 51,596 converted tyrosol to a lesser extent to compounds near icariside D2 with an Rt of 5.75 min (compound 3), while they did not show activity with hydroxyltyrosol; 35,728 and 48,377 converted tyrosol to salidroside and compound 3, respectively, while they could not react with hydroxyltyrosol; 43,413 did not show activity with tyrosol or hydroxyltyrosol; and 7146 converted tyrosol completely to an unknown compound (compound 4) with an Rt of 10.74 min, but could not catalyze hydroxytyrosol (Fig. 5). C4-glycosylation was not observed in the catalyzation of tyrosol or hydroxyltyrosol. The compound 1, 2, 3 and 4 were needed further analysis. Based on these results, we identified three UGTs (18,726, 35,728, and 48,377) with regio-specific T8GT activity. No GTs with region-specific T4GT activity were found in FLL.

## Discussion

Dry FLL is known as a phytomedicine and its plant is a greening plant in many countries. FLL is rich in various bioactive compounds, such as triterpenes, phenylethanoid glycosides, secoiridoids, and flavonoids. However, little is known about the dynamic changes of these main compounds, or their biosynthesis pathway as FLL ripens. Furthermore, considering the high price of olive oil [18], the scarcity of *Rhodiola* plants [19], and the large amount of FLL wasted every year, a clear understanding of the accumulation rules and molecular mechanism of the active components would be helpful in developing and utilizing FLL for use in food, medicine, and other fields. In this study, 70 compounds were identified across the six periods of FLL, and the main compounds, such as specneuzhenide, oleuropein and nuezhenoside G13, were quantified using HPLC. By combining transcriptome and metabolome analyses, changes in the expressions of genes and metabolites related to flavonoids, phenylethanols, triterpenoids, and secoiridoids were analyzed. The functions of C8-specific GTs in secoiridoids and phenylethanol biosynthesis were characterized using enzymatic activity assay.

Terpenoids, mainly secoiridoids and triterpenoids, are the major secondary metabolites of FLL, and they have a range of biological activities. Triterpenoids are enriched in FLL, reaching up to 4–5%, especially oleanolic acid, with contents ranging from 7.37 to 13.3 mg/g [1]. Because of this, FLL is the main source of oleanolic acid in the market [2]. In our study, the ion intensity of oleanolic acid was stable across the different periods. Oleanolic acid biosynthesis has been demonstrated due to its various pharmacological effects [20]. Oleanolic acid can be generated from 2,3-oxidosqualene, which is generated from FPS by SQS and SQE. Then, 2,3-oxidosqualene is processed by BAS and CYP716A to produce oleanolic acid. The genes related to the MEP and MVA pathways were tended to be expressed in the 45 DAF and 75 DAF period. A large number of precursors substance of terpenoids may have been accumulated in these two sampling times. *FPPS* was highly expressed from 45 DAF to 135 DAF, guiding the compounds flow to triterpenes. These downstream genes, such as *SQE, SQS, BAS*, and *CYP716A*, were expressed in all the stages, and may be the reason for the stable oleanolic acid content, which was consistent with previous reports [21].

Secoiridoids, such as those found in FLL and olives, are abundant in Oleaceae and are widely used in food, lumber, cosmetics, edible olive oil, and medicine [22]. Oleuropein, the most abundant compound in olive oil, was the first oleoside-type secoiridoids reported [10]. In FLL, elevated levels of oleuropein were observed during the immature fruit periods, specifically 45 DAF, 75 DAF, and 112 DAF, but decreased as the fruit underwent physiological development. These observations align with what has been observed in olives. The extent to which oleuropein degradation occurs may be influenced by the activity of *β*-glucosidase [4]. *β*-glucosidase activity may have been higher at 135 DAF and 170 DAF than in the immature fruit stages, so the oleuropein content in these two stages was lower. However, the lowest oleuropein content was observed in dry FLL. It is possible that the transition from fresh fruit to dry FLL may affect the stability of this compound. Oleuropein was also enriched in olive oil that was extra-virgin cold-pressed rather than extracted, probably because it is heat-unstable. Due to their similar structures, ligstroside was also likely to undergo degradation by *β*-glucosidase, resulting a significant accumulation of its content at 45 DAF, 75 DAF, and 112 DAF, followed by a substantial decrease in the subsequent 135 DAF, 170 DAF and 195 DAF. In addition, specnuezhenide and nuezhenoside G13 both share a common *β*-glucose structure and are susceptible to the activity of *β*-glucosidase. This enzymatic action may lead to a reduction in their contents at 170 DAF and 195 DAF. In contrast, these two compounds exhibited higher levels of enrichment in dry FLL (fully ripened and dried fruits) compared to fresh fruits at 170 DAF and 195 DAF. It is possible that the activity of *β*-glucosidase was inhibited during the drying process, resulting in the cessation of the conversion of these two products. Additionally, the activity of catalytic enzymes inside the fruit continued for a certain period of time after harvesting, which may have led to an increase in the levels of these two compounds in dry FLL. Thus, the changes in secoiridoids, in addition to environmental factors, are closely related to their biosynthesis.

The biosynthetic route for the formation of oleoside-type secoiridoids has only been partially reported [23]. Based on their structures, it seems that spenuezhenide, oleuropein, nuezhenoside G13, ligstroside, and neonuzhenide were produced from OME-glu esterized with tyrosol, hydroxytyrosol, or their glucosides. OME-glu is a glycosylated product of OME, which is known to mediate oleoside biosynthesis. Glycosylation and esterification have not yet been demonstrated and characterized [4, 11]. The quantitative analysis of OME and OME-glu showed that there was almost no OME-glu in dry FLL, but a small amount of OME remained. When the content of oleosides such as spenuezhenide and G13 were lower during the six periods, OME-glu also had a lower content, while OME had a higher content (Fig. 1). This variation in the contents of these compounds suggested that the conversion of OME to OME-glu was slow in the later periods (135 DAF to 195 DAF), while the consumption of OME-glu was rapid. During these stages, oleoside content was stable but OME-glu content increased slightly, suggesting that the GT activity of OME on OME-glu decreased gradually in the later harvesting times. To explore the specific GT of OME, we isolated the total FLL protein caused it to react OME. However, the expected chromatographic peak of OME-glu did not appear (Fig. S12). Compared with the deactivation protein, the peak area of OME obviously decreased in the total protein reaction. This may be because the total protein extract was more complex and contained other small-molecule compounds. OME-glu might have reacted rapidly and therefore would not have been detected. According to studies on plant acyltransferases, it is highly probable that enzymes with esterification activity belong to the serine carboxypeptidase acyltransferase (SPCL) family, which utilizes 1-*O*-*β*-glucose esters as acyl donors [24]. The structure of OME-glu can be recognized as a 1-*O*-*β*-glucose ester that react with tyrosol, although this has not yet been demonstrated. The esterification activity may also be performed using BAHD, which is named after the first four biochemically characterized ATs, namely benzylalcohol *O*-acetyltransferase, anthocyanin *O*-hydroxycinnamoyltransferase, anthranilate *N*-hydroxycinnamoyl/benzoyltransferase, and deacetylvindoline 4-*O*-acetyltransferase [24]. Hence, the biological function and biocatalytic potential of esterification and glycosylation warrant further study. These two unknown catalytic steps can potentially be elucidated based on the analysis of the complete genome. In addition, oleoside-type secoiridoids are predominantly found in the *Oleaceae* family, including plants such as *Osmanthus fragrans*, olive, and FLL. These plants belong to different genera and exhibit distinct geographical distribution characteristics, yet they have the capability to produce compounds with similar structures. The co-evolution of these plants within the secoiridoids biosynthetic pathway could potentially explain the occurrence of this phenomenon.

Oleoside-type secoiridoids, such as oleuropein and ligstroside, contain phenolic moieties such as tyrosol, hydroxytyrosol, and their C8-glycosylation salidroside and hydroxysalidroside. The production of these compounds is important for the biosynthesis of oleoside-type secoiridoids [15]. Tyrosine can react with TyDCs, MAO, and AAS to produce 4-HPAA. Because of the high amino acid sequence identity between TyDC and AAS, they cannot be separated using primary sequence analysis, so them were grouped for analysis [25]. There were two expression trends of *TyDC/AAS* in FLL; therefore, the functions of the sequences require further study. The specific glycosylation step of tyrosol has been explored in *R. rosea*. Genes homologous to *RrT4GT* and *RrT8GT* were cloned and investigated for their potential roles in the glycosylation of tyrosol and hydroxytyrosol (Fig. 5). The maximum likelihood (ML) tree of these homologs was consistent with their protein similarity (Fig. S7D). However, the T4GT homologs, such as Cluster-13715.51696, Cluster-13715.41413, and Cluster-13715.7146, did not have T4GT activities. In contrast, the T8GTs 35,728, 18,726, and 48,377 converted tyrosol to salidroside. 35,728, 18,726, and 48,377 were found to be highly expressed in our RNA-seq data (Fig. S7E). Among these, Cluster-13715.18726 has excellent activity, and can completely catalyze tyrosol and hydroxytyrosol. It is unfortunate that the proteins with esterification and glycosylation in the biosynthesis of oleoside-type secoiridoids are still unknown. In order to investigate the biosynthesis of oleosides and characterize the specific enzymes involved, a better understanding of the transition of the compounds between oleosides is essential. This knowledge will enable the reconstitution and optimization of oleoside metabolic pathways, and lays the foundation for the comprehensive development and utilization of FLL to utilize its health-promoting properties. The results of this study provide a basis for reasonable selection of harvest time of FLL, and also provide a foundation for a comprehensive use and development of FLL resources.

## Conclusions

In this study, transcriptomic and metabolomic analyses revealed the rules that describe the dynamic changes in the compounds comprising FLL across development periods. Metabolomic data identified 70 compounds across development periods, most of which were secoiridoids. Eleven other compounds were also quantified, such as tyrosol, salidroside, specnuezhenide, oleuropein, G13. The marker compound specnuezhenide was significantly upregulated at 112 DAF, and G13 was enriched at 135 DAF, the coloring period. Oleuropein was abundant at 45 DAF when FLL was in the young fruit stage. Using transcriptome data, we analyzed the biosynthetic pathways of the main components of FLL. In secoiridoid biosynthesis, gene expression was similar between *SXS/OMES* and *ISY*, suggesting that they may be co-expressed that the upregulation of these genes may be beneficial for oleuropein biosynthesis. The in vitro enzyme activity of GTs, similar to T4GT and T8GT, showed their catalytic ability to convert tyrosol and hydroxytyrosol to salidroside and hydroxysalidroside, respectively. Therefore, this study provides valuable information about the harvest period of FLL according to how the compounds accumulate. It also lays foundation for the molecular research on the improvement of FLL quality.

## Materials and methods

### Plant materials and standards

The samples in the present study were harvested from plants at Nanjing University of Chinese Medicine (Nanjing, China), which were identified by the corresponding author of this article (Professor Qinan Wu). Three fruit trees with good growing conditions at the same developmental period were selected, and sampling of fruits was carried out on August 16, 2021 (45 days after flowering, DAF), September,16 2021 (75 days after flowering), October 23, 2021 (112 days after flowering when the color of the exocarp began to change), November 16, 2021 (135 days after flowering), December 16, 2021 (170 days after flowering when the day of the Winter Solstice Festival in China is known to be the best time for harvesting), and January 16, 2022 (195 days after flowering) (Fig. S1). The six sampling times were named as 45 DAF, 75 DAF, 112 DAF, 135 DAF, 170 DAF, 195 DAF, respectively. The harvested samples were quickly frozen in liquid nitrogen and stored at -80℃. The dry FLL served as the standard traditional Chinese medicine which was used as control. Three biological replicates were used for each experiment.

Specnuezhenide, oleanolic acid, ursolic acid, salidroside, tyrosol, neonuezhenide, ligustroflavone, nuezhenoside G13, oleoside-11-methyl ester (OME), and oleuropein standards were purchased from Chengdu DeSiTe Biological Technology (Chengdu, China). Hydroxysalidroside and hydroxytyrosol ligstroside were purchased from Shanghai Yuanye Bio-Technology (Shanghai, China), and 7-*β*-1-D-glucopyranosyl-11-methyl oleoside (OME-glu) was purchased from MolPort (Riga, Latvia). HPLC-grade acetonitrile was purchased from Merck (Germany), and MS-grade formic acid was purchased from Sigma-Aldrich (Germany). All other chemicals and solvents used were of analytical grade. Ultrapure water was purchased from Watsons (Hongkong, China). The standards were all dissolved in methanol.

### Sample preparation for LC-MS

The biological samples were freeze-dried using a vacuum freeze-dryer (Scientz-100 F). The freeze-dried samples were crushed using a mixer mill (MM 400, Retsch) with zirconia beads for 1.5 min at 30 Hz. Then, 0.3 g lyophilized powder was added to 10 mL 80% methanol solution and treated with ultrasound for 30 min three times. After centrifugation at 12,000 g for 10 min, the extracts were filtered through a 0.22-µm membrane filter before LC-MS/MS analysis. There were six biological repeats in each group.

The metabolite profiles were analyzed using a triple TOF 5600 + system (AB SCIEX, Foster City, CA, USA) with an ESI source coupled to a UPLC system (Shimadzu 30 A UHPLC system, Shimadzu, Japan). For UPLC analysis, a 2 µL sample was injected into an analytical reverse-phase column (XBridge C18 Column 150 mm × 4.6 mm, 3.5 μm). Separation was performed with 0.1% formic acid in water (A) and 0.1% formic acid in acetonitrile (B). The total running time was 45 min and the flow rate was 0.6 mL/min. The column compartment was maintained at 30 °C. The mobile phase had a gradient elution of 5 − 20% B (0–5 min), 20 − 35% B (5–21 min), 35 − 70% B (21–22 min), 70 − 97% B (22–38 min), 97% − 5% B (38–42 min) and 5% B (42–45 min). For TOF analysis, the mass spectrometer was operated in the positive and negative ESI modes with a duo-spray source, and the mass scan range was set at *m*/*z* 50-1500 for both TOF-MS and TOF-MS/MS scans. The detailed parameters used were as described previously [26].

### Quantitative analysis

A Waters Acquity HPLC™ system (e2695; Waters Corp., MA, USA) equipped with a TUV detector system (2998; Waters Corp.) was used for the quantitative analysis of specnuezhenide, salidroside, nuezhenoside G13, hydroxysalidroside, oleuropein, ligstroside, ligustroflavone, OME, and OME-glu. Chromatography was performed on an XBridge C18 column (250 m × 4.6 mm, 5 μm). The mobile phase consisted of acetonitrile (A) and 0.15% formic acid in water (B). The HPLC elution conditions were optimized as follows: 0–20 min, 10 − 18% A; 20–30 min, 18 − 35% A; 30–52 min, 35 − 69% A; 52–60 min, 69% − 10% A; 60–65 min, 10% A. The flow rate was 1.0 mL/min, the column was maintained at 28 ℃, the injection volume was 10 µL, and the detection wavelength was set at 253 nm. Based on a previous method, an external calibration method was used for the quantitative analysis [9]. Linear calibration curves were constructed for specnuezhenide (y = 4778.2x + 5653.0), salidroside (y = 2415.1x + 1912.0), nuezhenoside G13 (y = 3649.0x + 3192.3), hydroxysalidroside (y = 738.22x − 234.77), oleuropein (y = 6978.6x + 5503.8), ligstroside (y = 5000.3x + 1726.9), ligustroflavone (y = 6629.5x − 220.73), OME (y = 6829.9x + 4991.3), and OME-Glu (y = 2276.5x + 8697.5). Good linear correlation and high sensitivity under these chromatographic conditions were confirmed using correlation coefficients (*R* > 0.999) (Table S1). The extracts used for LC-MS analysis were injected into an HPLC system for the quantitative analysis of the chemicals mentioned above. The data are expressed as mean values with standard deviation and were obtained from six biological replicates.

### RNA extension and transcriptome analysis

Total RNA of fruit samples harvested at different times was extracted using the CTAB reagent (QIAGEN, Germany). RNA samples were separately sequenced using an Illumina NovaSeq 6000 platform (Illumina, USA) following the manufacturer’s recommendations, in which the mRNA was purified using poly-T oligo-attached magnetic beads. Library quality was evaluated using an Agilent 2100 bioanalyzer. After each library was qualified, the different libraries were pooled according to the effective concentration and the target amount of data from the machine and then sequenced using an Illumina NovaSeq 6000 system (Illumina). There were three biological repeats in RNA-Seq.

### Assembly, annotation, and differential gene expression analysis

Raw data were cleaned by removing low-quality reads, adapter reads, and N-containing reads. Trinity software (v2.6.6) was used to assemble the clean reads. Annotation was performed using the following databases: Nr, Nt, Pfam, COG/KOG (Clusters of Orthologous Groups of proteins), Swiss-Prot, KEGG, and GO. Differential expression analyses of the different development periods were performed using the DESeq2 R package (v1.20.0). The resulting *P*-values were adjusted using Benjamini and Hochberg’s approach to control the false discovery rate. *P*_adj_ < 0.05 and |log2(foldchange)| > 1 were set as thresholds for significantly differentially expressed genes. Based on the results of the analyses, GOseq (v1.10.0) and KOBAS (v2.0.12) software were used for GO function enrichment analysis and KEGG pathway enrichment analysis of the differential gene sets.

### Quantitative real-time PCR analysis

The qRT-PCR analysis was performed using a QuantStudio 3 Real-Time PCR System (Thermo Fisher Scientific Inc., Waltham, MA, USA) and a ChamQ Universal SYBR qPCR kit (Vazyme Biotech, Nanjing, China). The amplification procedure and qRT-PCR reaction solution was performed as previously described [24], with three biological replicates. *ACTIN* was used as the internal reference gene. Finally, relative gene expression was calculated using the 2^−∆∆Ct^ method. All primers used for the qRT-PCR analyses are listed in Table S2.

### Gene identification

The candidate genes in the biosynthesis were firstly selected using their KEGG and gene annotations; then each gene was blasted in NCBI manually. Cr7DLH (AGX93062.1), CrLAMT (AGX93063.1), CrSLS (AAA33106.1), Oe7eDLH (ALV83449.1), OeOMES (QOW17548.1), OeSXS (QOW17550.1), and OeLAMT (AFS28695.1) were used to search for homologous genes in FLL. The T4GTs and T8GTs of FLL were homologous genes of RrT4GT (AUI41117.1) and RrT8GT (AUI41147.1), respectively. RrT4GT and RrT8GT were set as query sequences, and FLL proteins were set as subject sequences with an E-value of 10 − 5. Sequences whose weight coverage was > 0.99 and with a subject_evalue of zero were selected, then the sequence lengths were checked. Sequences with similar lengths and having the same conserved domain were reserved. These sequences were then used in an enzymatic activity assay. The maximum likelihood (ML) tree of the T4GTs and T8GTs used MEGA X software (Mega Software, Dortmund, Germany) with 1,000 bootstrap-based JTT + G + I amino acid substitution models.

### Gene isolation, enzyme expression, and enzymatic activity assay

A HiScript III 1st Strand cDNA Synthesis Kit (+ gDNA wiper) (Vazyme Biotech) was used with 0.5 µg of total RNA to synthesize the first-strand cDNA, which was used as the template for PCR with a 2 × Taq Master Mix (Vazyme Biotech). Gene-specific primers were designed based on annotated results from the transcriptome database. The PCR products were separated using a GeneJETTM Gel Extraction Kit (Thermo Fisher Scientific), and the purified DNA fragments were cloned into a pET28a vector using a ClonExpress II One Step Cloning Kit (Vazyme Biotech). All clones were sequenced using Sanger sequencing (Tsingke, Beijing, China). The recombinant vector was transformed into *Escherichia coli* BL21 cells (TIANGEN, Beijing, China) using the heat shock treatment method at 42 ℃ for 45 s. Finally, transformants were plated on LB medium containing 100 ng/L kanamycin.

Single colonies were used to inoculate into LB medium (5 mL) containing 100 ng/L kanamycin. The culture (1 mL) was transferred to 100 mL of the same medium and continued to grow at 37 °C to reach an OD_600_ of approximately 1.0. Protein expression was induced by adding isopropyl *β*-d-thiogalactopyranoside at a final concentration of 1.0 mM. After incubation at 18 ℃ for 20 h, the cells were harvested at 5,000 g, 4 ℃ for 15 min and resuspended in 2 mL lysis buffer (15% *v*/*v* glycerinum, 10 mM Tris-HCl [pH 8.0], and 200 mM NaCl). The suspension was then transferred to an ultrasonic cell disruptor system and ultrasonically disrupted for 25 s each, with 35 s on ice between disruptions with three times. The mixture was then centrifuged (30 min, 4 °C, 12,000 g), and the supernatant was carefully collected as the crude protein.

The reaction system of enzyme activity in vitro is as follows: the crude protein (40 µL), 50 mM Tris-HCl (pH 7.5), 500 µM substrate, 2 mM UDPG. They were added to a 250 µL reaction system, and incubated at 30 ℃ in the dark on a shaker. The reaction was stopped by adding 250 µL of methanol. Next, all samples were centrifuged at 12,000 g for 10 min and, the supernatant was subjected to HPLC analysis.

The HPLC system used was the same as that for the quantitative analysis. Chromatography was performed on an XBridge C18 column (250 m × 4.6 mm, 5 μm). The mobile phase consisted of acetonitrile (A) and 0.1% formic acid in water (B). The HPLC elution conditions were optimized as follows: 0–8 min, 10- 20% A; 8–10 min, 20 − 40% A;10–13 min, 40 − 70% A; 13–16 min, 70 − 95%; 16–19 min, 95%; 19–21 min, 95% − 10%; 21–25 min, 10%. The flow rate was 0.7 mL/min, the column was maintained at 30 ℃, and the injection volume was set as 10 µL.

### Electronic supplementary material

Below is the link to the electronic supplementary material.

Supplementary Material 1

## Data Availability

The datasets generated and/or analyzed during the current study are available in the Sequence Read Archive (SRA repository), under the Accession number PRJNA874863 [https://www.ncbi.nlm.nih.gov/bioproject/PRJNA874863] at NCBI. The RNA-seq data has been submitted to NCBI SRA: PRJNA874863 (The data will be available after the study is published).

## Abbreviations

FLL: Fructus Ligustri Lucidi
TCM: traditional Chinese medicine
7eDLH: 7-deoxy-loganic acid 7-*epi*-hydroxylase
OMES: oleoside methyl ester synthase
SXS: secoxyloganin synthase
GT: glycosyltransferase
HPLC: High Performance Liquid Chromatography
MVA: mevalonic acid
MEP: methylerythritol phosphate
DMAPP: dimethylallyl diphosphate
AACT: acelyl-CoA *C*-acetyltransferas
HMGCS: hydroxymethylglutaryl-CoA synthase
HMGCR: hydroxymethylglutaryl-CoA reductase
MVK: mevalonate kinase
PMK: phosphomevalonate kinase
DXS: 1-deoxy-D-xylulose-5-phosphate synthase
DXR: 1-deoxy-D-xylulose-5-phosphate reductase
MCT: 2-*C*-methyl-D-erythritol 4-phosphate cytidylyltransferase
CMK: 4-diphosphocytidyl-2-*C*-methyl-D-erythritol kinase
MDS: 2-*C*-methyl-D-erythritol 2,4-cyclodiphosphate synthase
HDS: (E)-4-hydroxy-3-methylbut-2-enyl-diphosphate synthase
HDR: 4-hydroxy-3-methylbut-2-en-1-yl diphosphate reductase
FPPS: farnesyl pyrophosphate synthase
SQE: squalene monooxygenase
SQS: squalene synthase
GES: geraniol synthase
GPP: geranyl diphosphate
G10H: geraniol 10-hydroxylase
10GHO: 10-hydroxygeraniol oxidoreductase
ISY: iridoid synthase
IO: iridoid oxidase
7DLGT: 7-deoxyloganetic acid glucosyltransferase
7DLH: 7-deoxyloganic acid 7-hydroxylase
LAMT: loganic acid *O*-methyltransferase
SLS: secologanin synthase
TyDC: Tyrosine/Dopa decarboxylase
4-HPAA: 4-hydroxyphenylacetaldehyde
3,4-DHPAA: 3,4 dihidroxyphenylacetaldehyde
MAO: monoamino oxidase
PAR: phenylacetaldehyde reductase
T8GT: UDP-glucose 8-*O*-glucosyltransferase
AAS: aromatic aldehyde synthase
T4GT: UDP-glucose 4-*O*-glucosyltransferase
F3H: flavanone 3-hydroxylase
F3’H: flavonoid 3′-hydroxylase
DFR: dihydroflavonol 4-reductase
ANS: anthocyanidin synthase
UFGT: UDP-glucose: flavonoid 3-*O*-glucosyltransferase
SPCL: serine carboxypeptidase acyltransferase
DAF: days after flowering
