# Supplementary material for: Transcriptome and metabolome analysis revealed the dynamic change of bioactive compounds of Fructus Ligustri Lucidi
Source: BMC Plant Biol. 2024 Jun 3;24:489. doi: 10.1186/s12870-024-05096-3 (PMC11145772; doi:10.1186/s12870-024-05096-3)
Supplement: Supplementary file 1 — Supplementary Material 1 [file 12870_2024_5096_MOESM1_ESM.pdf]

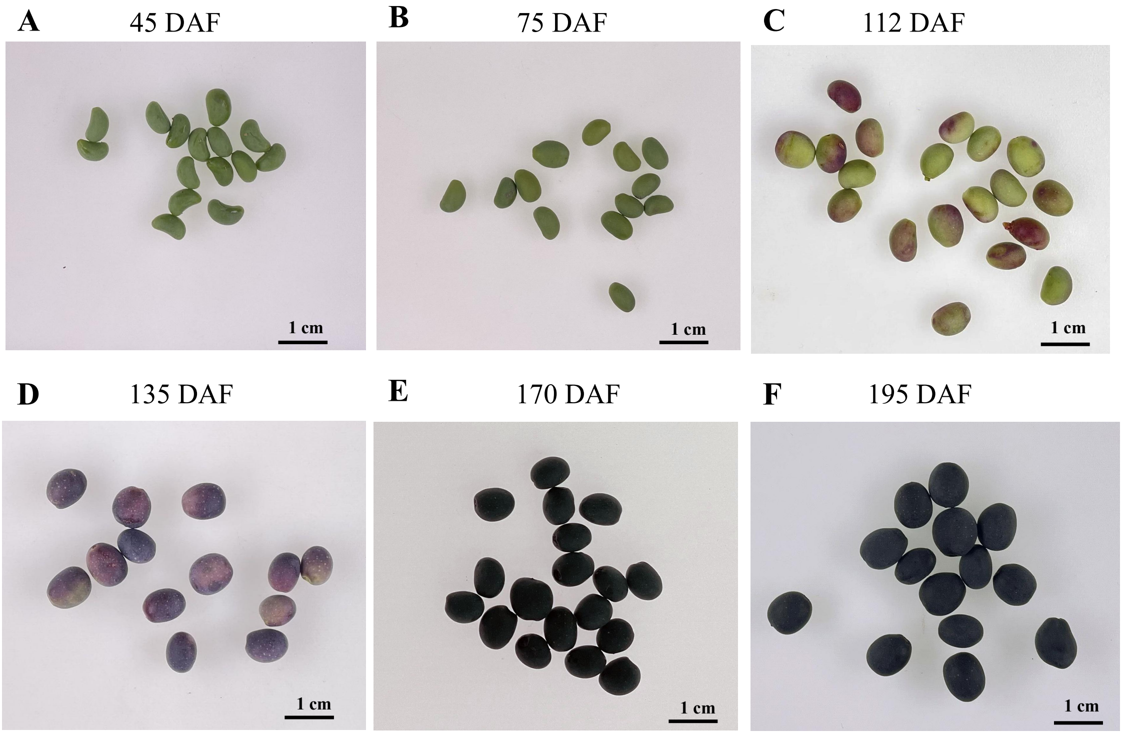

Fig. S1 Phenotype of FLL at six fruit development periods. Scale bar = 1 cm.

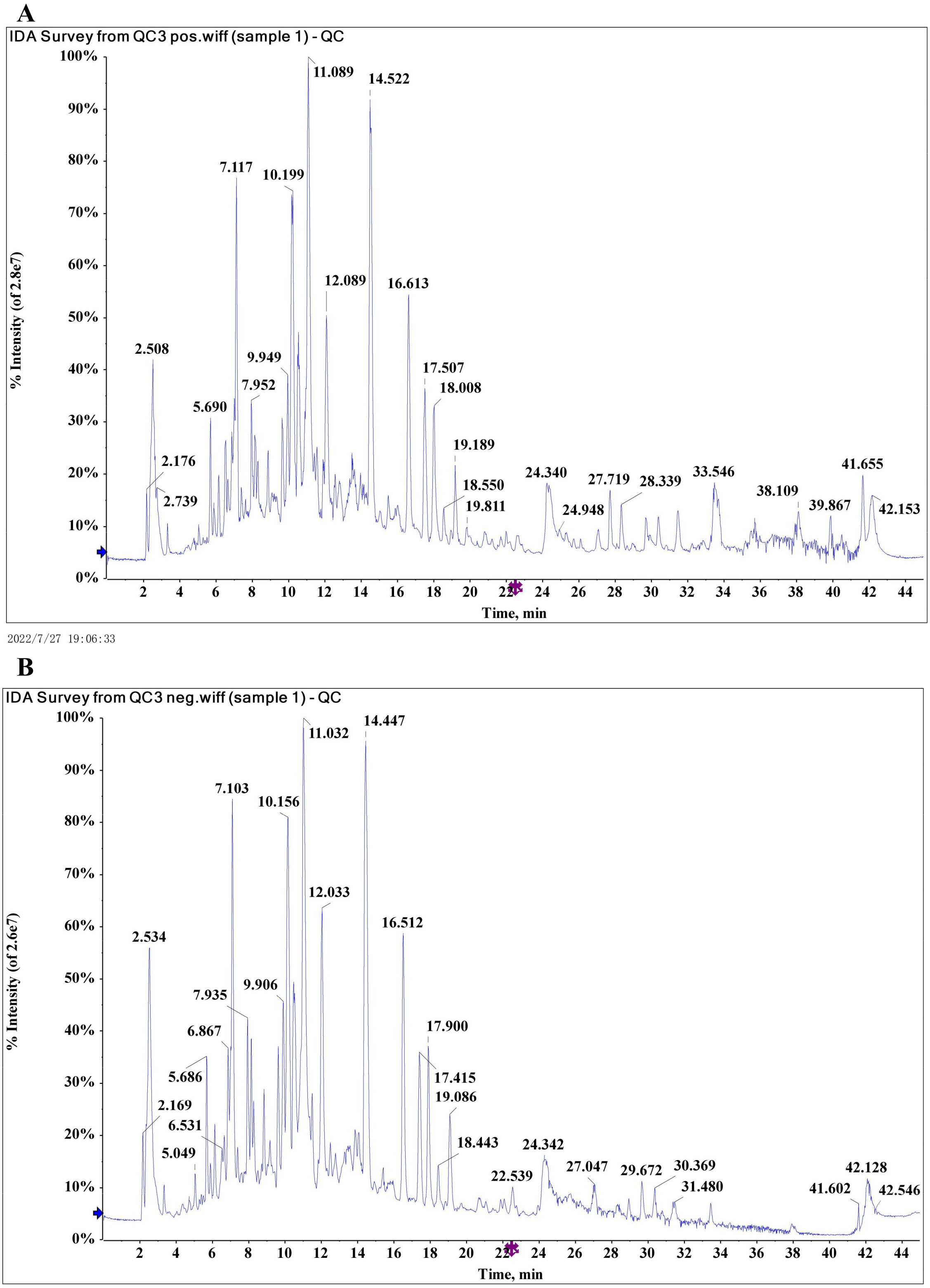

Fig. S2 The TIC chromatograms of FLL extracts for postive (A) and negative (B) mode.

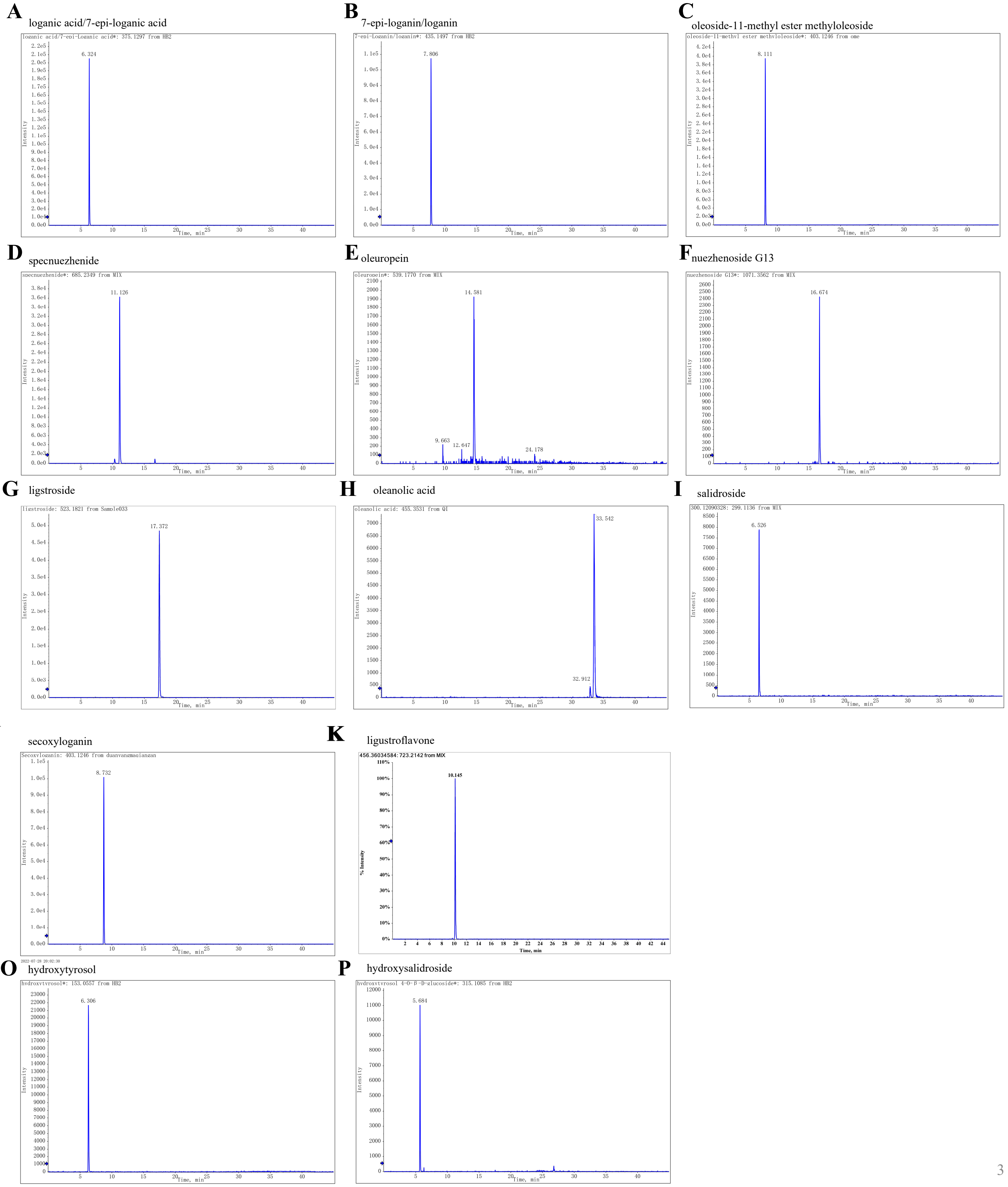

Fig. S3 The MS/MS of compounds identified by standards.

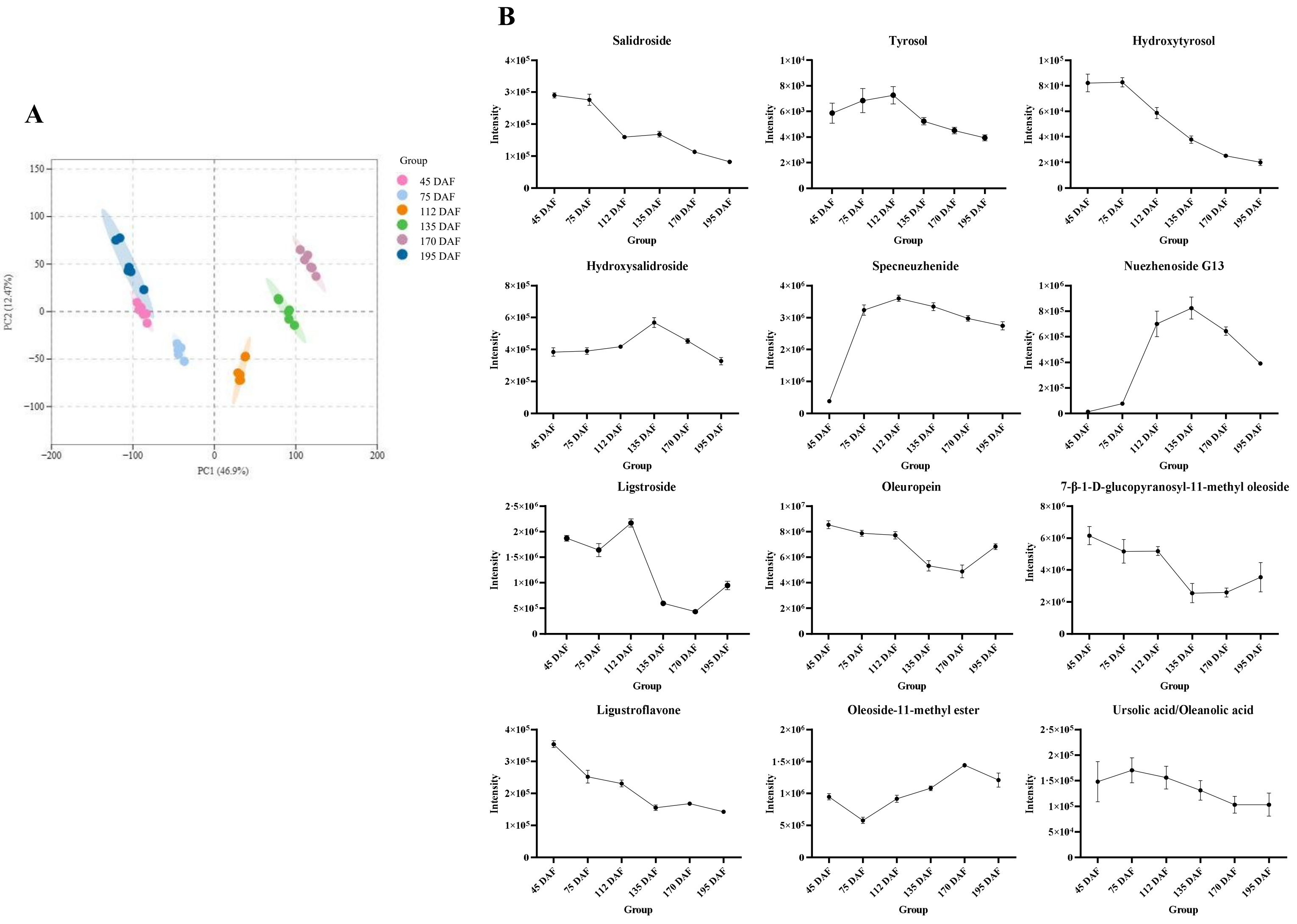

Fig. S4 Principal component analysis (PCA) of all identified metabolites in six fruit development periods (A); The trends of intensity of 12 main compounds (B)

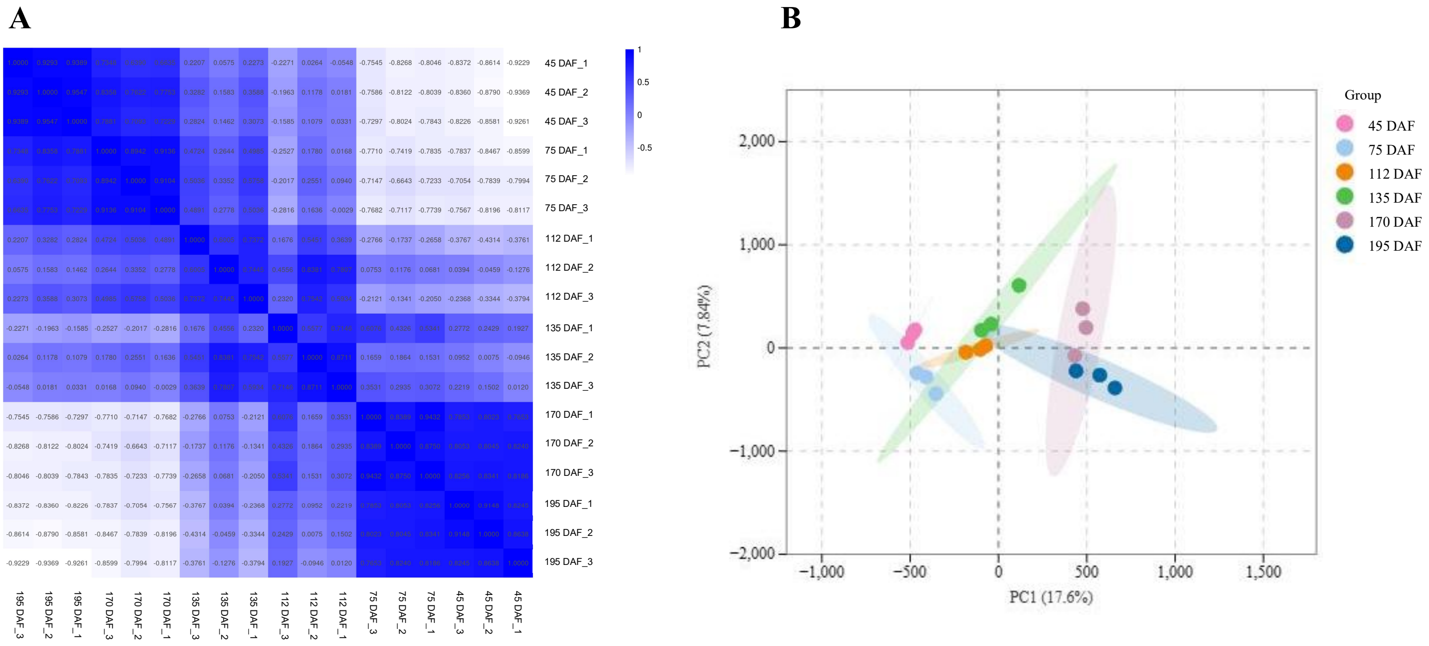

Fig. S5 An inter-sample correlation analysis of RNA-seq on the 18 samples among six fruit development periods (A) and PCA of these samples (B).

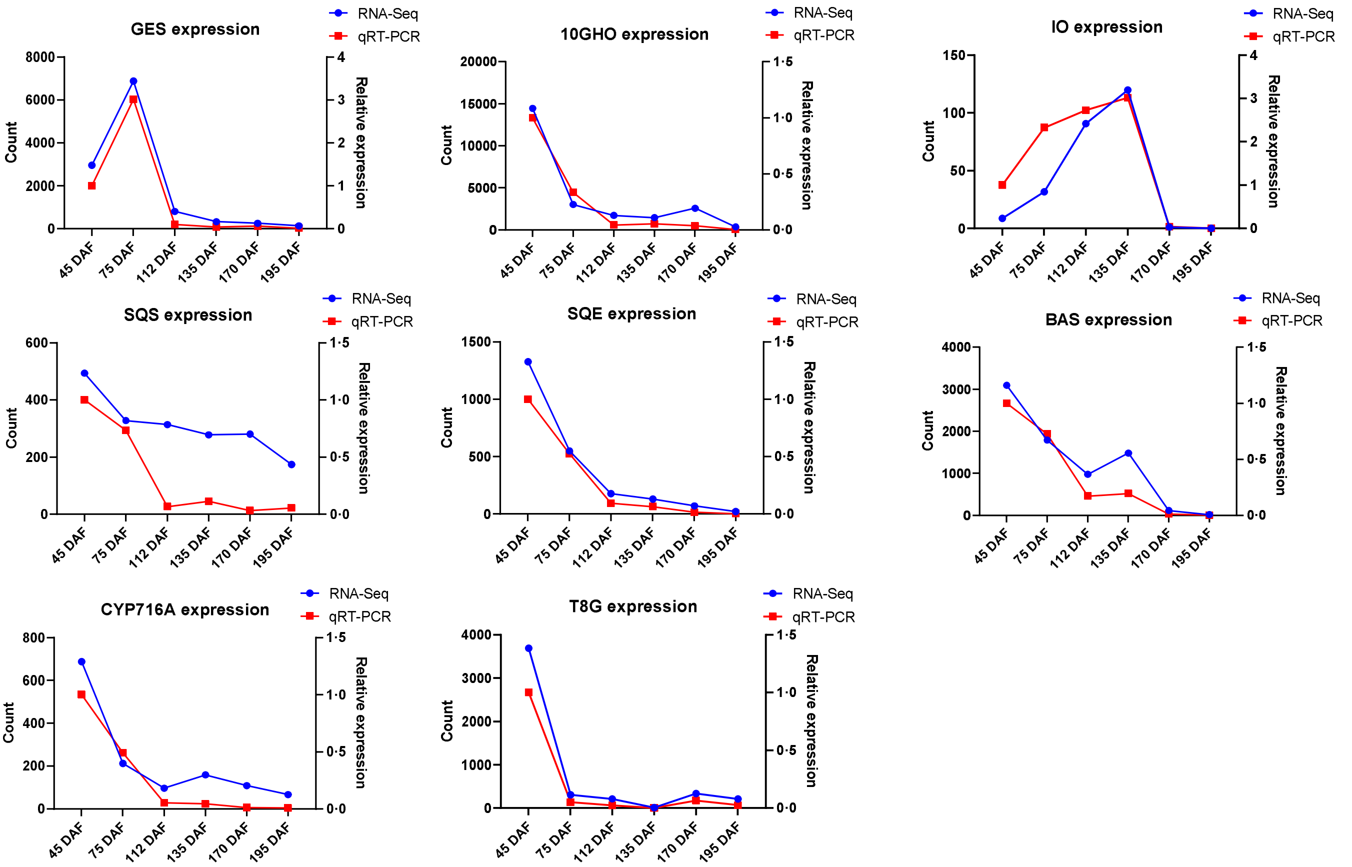

Fig. S6 The expression level in RNA-Seq and qRT-PCR of representative genes involved in oleanolic acid biosynthesis, secoiridoids biosynthesis, and salidroside biosynthesis.

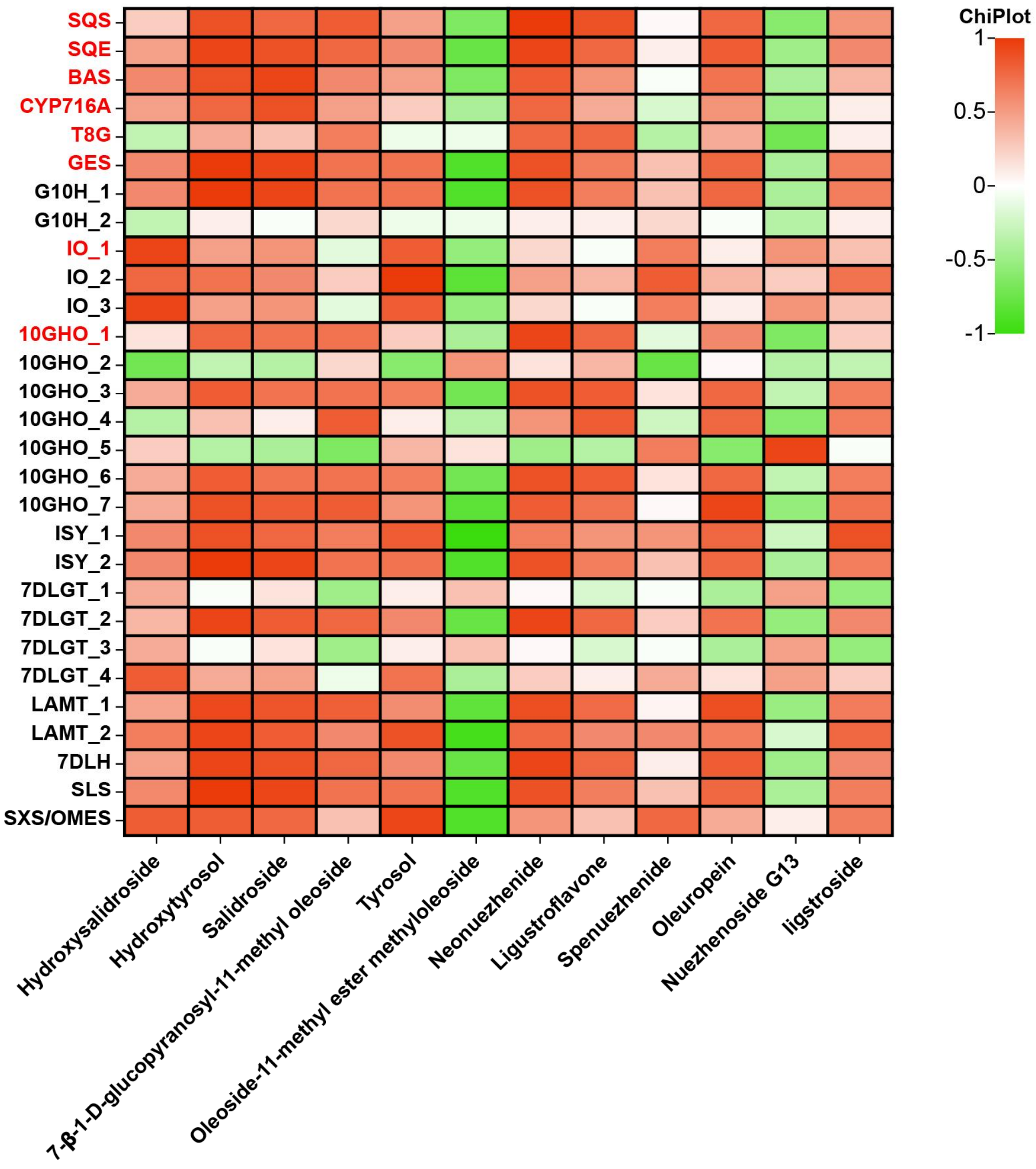

Fig. S7 The correlation between gene expression and quantitative compounds contents involved in oleanolic acid biosynthesis, secoiridoids biosynthesis, and salidroside biosynthesis. The genes performed by qRT-PCR were marked with red.

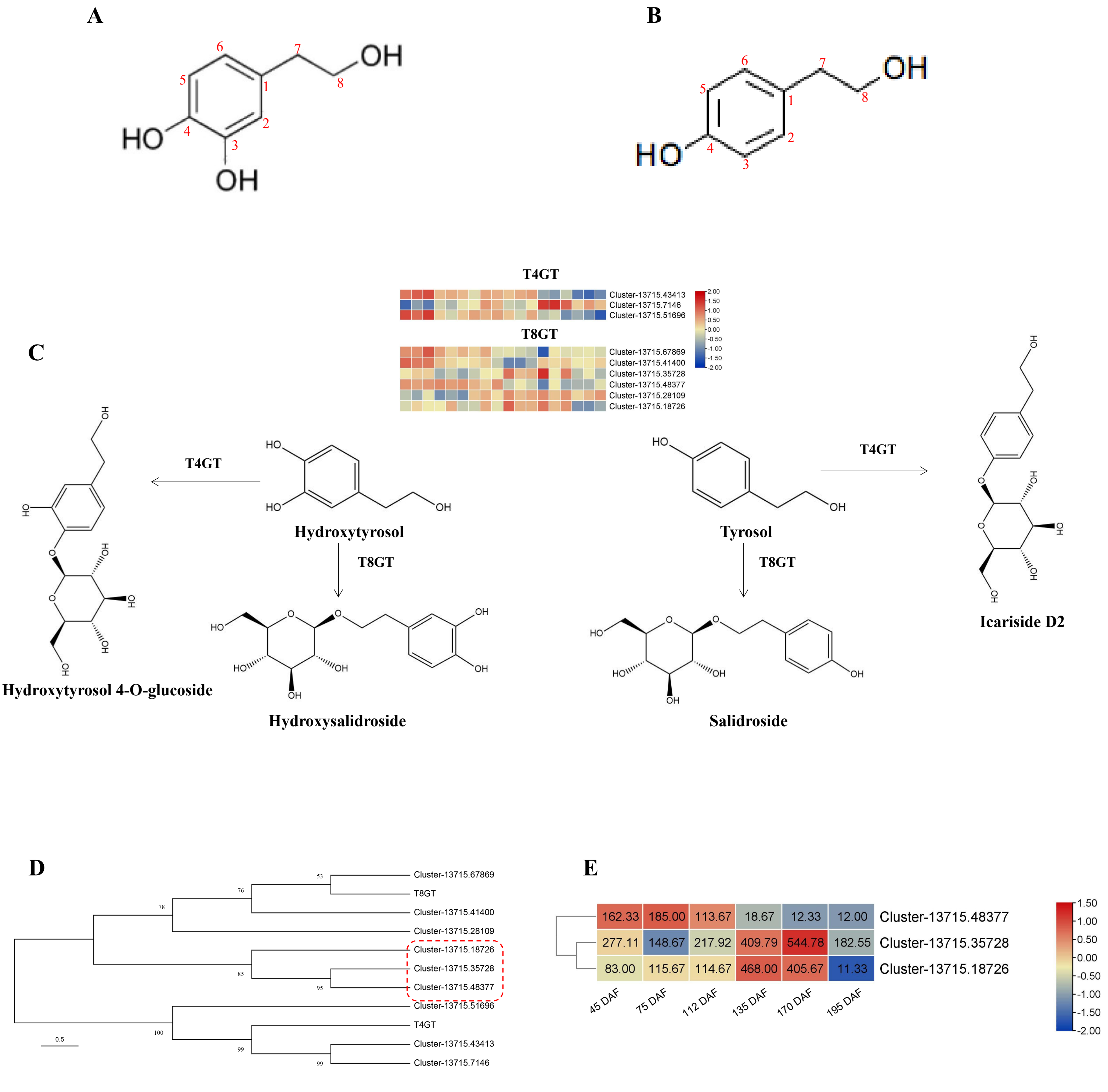

Fig. S8 The structures of hydroxyltyrosol (A) and tyrosol (B). The biosynthesis of salidroside and hydroxysalidroside and related gene expression (C). Maximum likelihood (ML) tree tof T4GTs and T8GTs (D). The expression of genes with C8-region glycosylation.

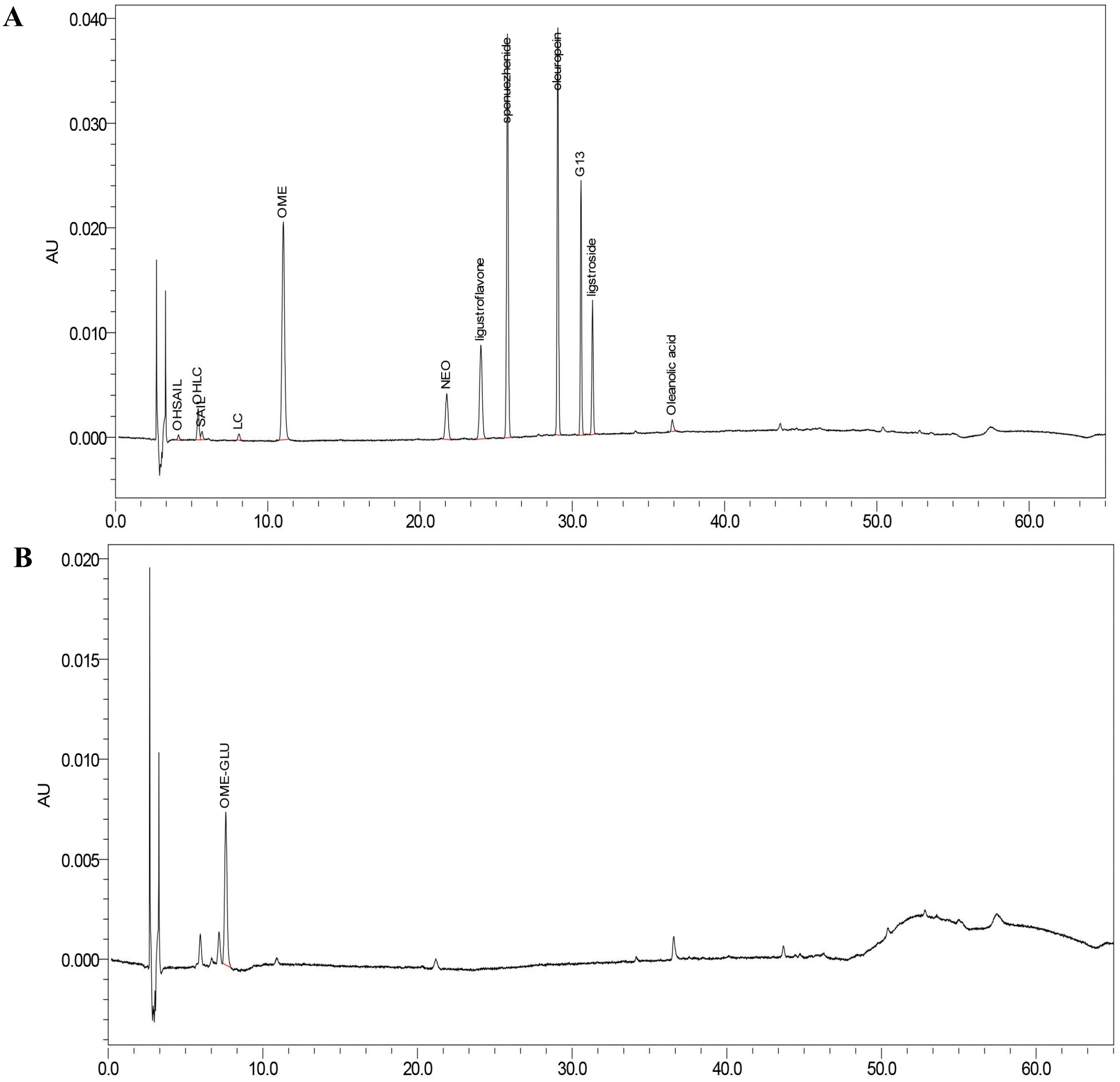

Fig. S9 The HPLC chromatograms of standard for quantified compounds.

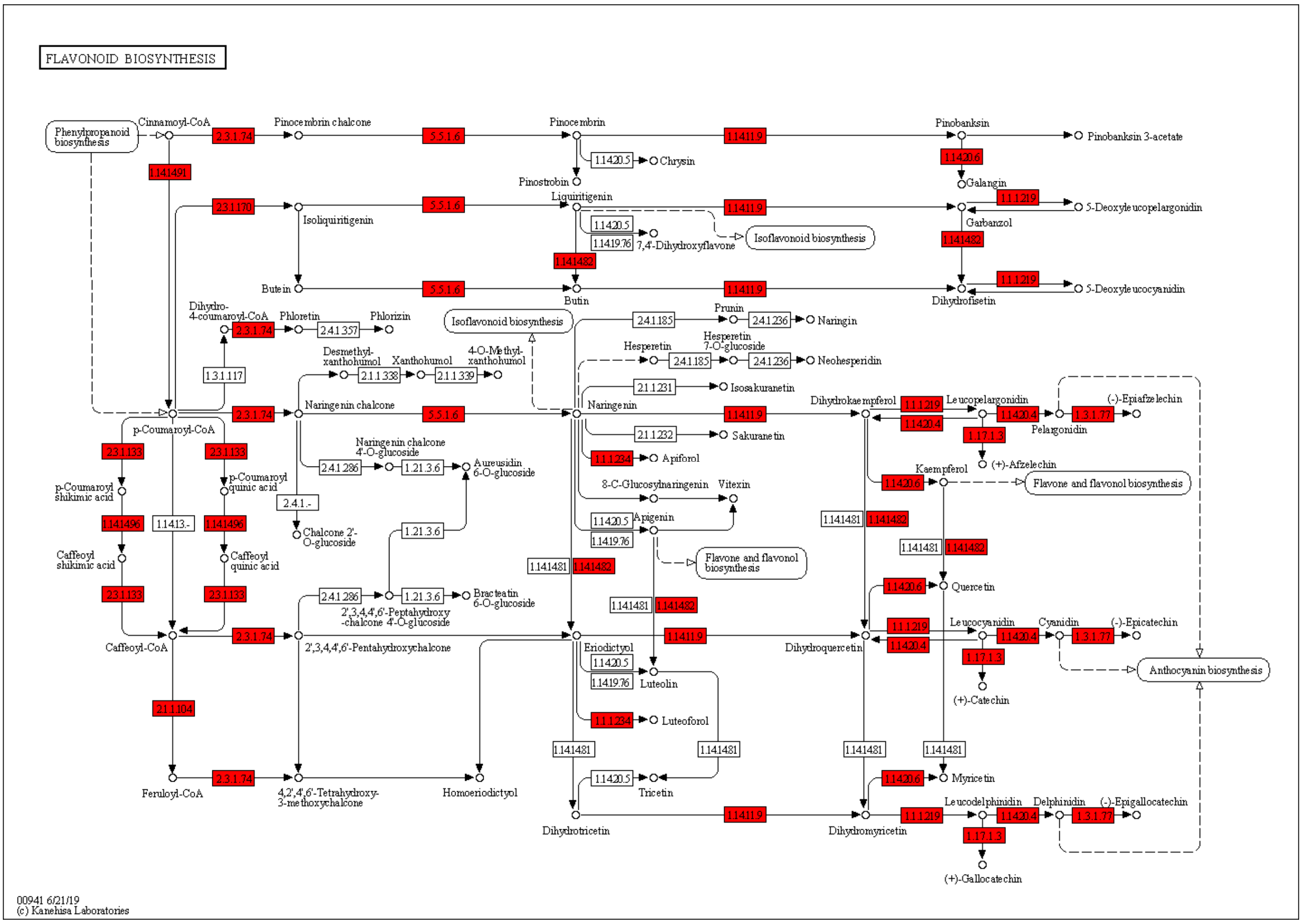

Fig. S10 The flavonoids biosynthesis in KEGG with mapped DEGs marked with red.

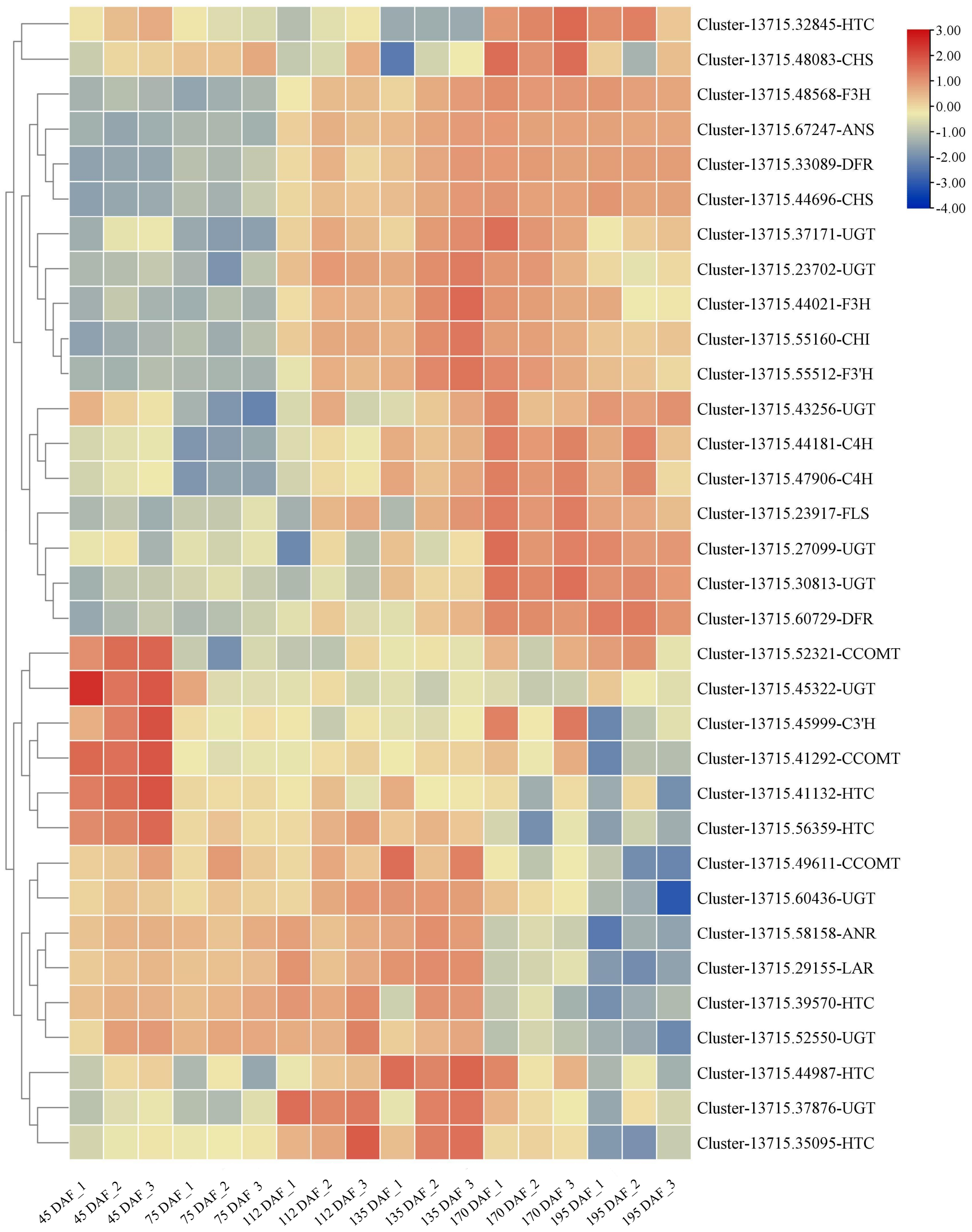

Fig. S11 The DEGs expression related to flavonoid biosynthesis during six fruit development periods of FLL.

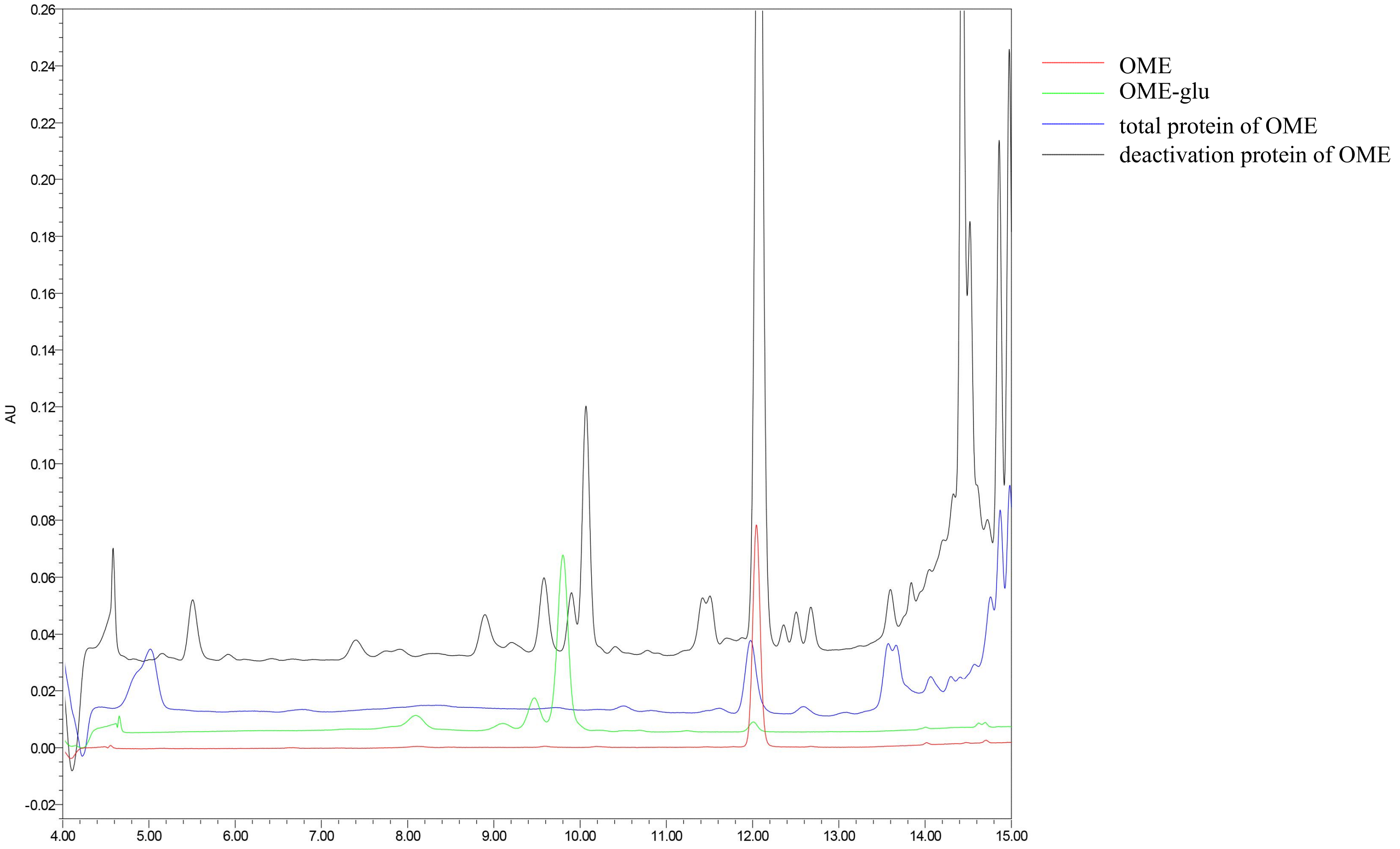

Fig. S12 HPLC chromatograms of products from total protein and deactivation protein with OME.

Table S1
The Linear equation of 9 compounmds.

Compound	Calibration curve	*R*	Linear range (ug/mL)
specnuezhenide	y=5653.0+4778.2x	1.0000	6.0-1900.0
oleuropein	y=5503.8+6978.6x	0.9999	3.6-1000.0
nuezhenoside G13	y=3192.3+3649.0x	0.9999	3.6-1400.0
ligstroside	y=1726.9+5000.3x	0.9998	1.6-150.0
ligustroflavone	y=-220.7+6629.5x	0.9996	1.6-30.0
oleoside-11-methyl ester	y=4991.3+6829.9x	0.9998	3.6-150.0
7-β-1-D-glucopyranosyl-11-methyl oleoside	y=8697.5+2276.5x	0.9993	10.8-3.6
tyrosol	y=-246.7+4482.1x	0.9993	0.8-8.0
salidroside	y=1912.0+2415.1x	0.9998	1.6-12.8

Table S2
The primers of qRT-PCR.

Gene	Primer-F	Primer-R
SQS	AGGAGAACTCGGTCAAGGCTGTAC	TCTACCTGTGGAATCGCATGAAAGC
SQE	TTCTGGGTTTGCTCTTACCATGTGG	GAGGGCAGGCTTGAATCCGAAG
BAS	CAGTTACCAGAGGCCGCAAGTG	CCGTCCCAATCATACACACCAAGG
GES	AGGCACTTCAGGATGGCTAGGTTAG	AAGGGCTGGATCGTGTTCACTTTC
10GHO	CGGAGTGGTAACGGAGATTGGAAAC	CGACATGATCCGACCAAGCATCC
IO	GGTCAACACTGGCGGATGCTAC	CACACCGTCGTCGTACAACTCG
T8GT	TTCTTCGCCGAGCAACAGACTAAC	TCCACTCCATCCCTCTTGACATCAC
CYP716A	ACTTCATCCAAAGAAGAGGCCATTAAGA	GTGTATCTATTTGCAAGAGGGAAAACTTTTACTTCA

Table S3

The basic information of transcriptomes.

sample	library	raw_reads	raw_bases	clean_reads	clean_bases	error_rate	Q20	Q30	GC_pct
45 DAF_1	FRAS220035915-1r	2.02E+07	6.1	1.96E+07	5.9	0.03	97.55	92.69	43.24
45 DAF_2	FRAS220035916-1r	2.18E+07	6.5	2.07E+07	6.2	0.03	97.88	93.66	43.36
45 DAF_3	FRAS220035917-1r	2.21E+07	6.6	2.16E+07	6.5	0.03	97.83	93.5	43.22
75 DAF_1	FRAS220035918-1r	2.02E+07	6.0	1.94E+07	5.8	0.03	97.61	92.9	43.13
75 DAF_2	FRAS220035919-1r	2.16E+07	6.5	2.09E+07	6.3	0.03	97.82	93.51	43.28
75 DAF_3	FRAS220035920-1b	2.28E+07	6.8	2.17E+07	6.5	0.03	97.95	93.62	42.15
112 DAF_1	FRAS220035921-1r	2.24E+07	6.7	2.09E+07	6.3	0.03	97.77	93.34	43.23
112 DAF_2	FRAS220035922-1r	2.13E+07	6.4	1.96E+07	5.9	0.03	97.79	93.39	42.64
112 DAF_3	FRAS220035923-1r	2.25E+07	6.8	2.15E+07	6.4	0.03	97.69	93.16	44.04
135 DAF_1	FRAS220035924-1r	2.12E+07	6.4	2.03E+07	6.1	0.03	97.58	92.89	43.10
135 DAF_2	FRAS220035925-1r	2.16E+07	6.5	2.07E+07	6.2	0.03	97.66	93.04	43.66
135 DAF_3	FRAS220035926-1r	2.22E+07	6.7	2.10E+07	6.3	0.03	97.68	93.07	43.34
170 DAF_1	FRAS220035927-1r	2.20E+07	6.6	2.09E+07	6.3	0.03	97.7	93.18	43.24
170 DAF_2	FRAS220035928-1r	2.08E+07	6.2	1.98E+07	6.0	0.03	97.62	92.92	42.77
170 DAF_3	FRAS220035929-1r	2.24E+07	6.7	2.16E+07	6.5	0.03	97.59	92.89	43.18
195 DAF_1	FRAS220035930-1r	2.21E+07	6.6	2.12E+07	6.4	0.03	97.54	92.82	43.19
195 DAF_2	FRAS220035931-1r	2.17E+07	6.5	2.10E+07	6.3	0.03	97.73	93.29	42.73
195 DAF_3	FRAS220035932-1r	2.24E+07	6.7	2.13E+07	6.4	0.03	97.71	93.15	41.67

Table S4

The genes annotation of databases.

Details	Number of Unigenes	Percentage (%)
Annotated in NR	49,472	48.00
Annotated in NT	46,843	45.45
Annotated in KO	15,831	15.36
Annotated in SwissProt	33,517	32.52
Annotated in PFAM	33,112	32.12
Annotated in GO	33,109	32.12
Annotated in KOG	8,499	8.24
Annotated in all Databases	5,047	4.89
Annotated in at least one Database	61,563	59.73
Total Unigenes	103,058	100

Table S5
The DEGs statistics of comparison.

Compare	All	Up	Down	Threshold
75 DAF vs 45 DAF	5,854	2,164	3,690	DESeq2 padj<0.05 |log2FoldChange|>1
112 DAF vd 45 DAF	8,323	3,250	5,073	DESeq2 padj<0.05 |log2FoldChange|>1
135 DAF vs 45 DAF	10,540	4,112	6,428	DESeq2 padj<0.05 |log2FoldChange|>1
170 DAF vs 45 DAF	19,404	8,143	11,261	DESeq2 padj<0.05 |log2FoldChange|>1
195 DAF vs 45 DAF	22,746	10,121	12,625	DESeq2 padj<0.05 |log2FoldChange|>1
112 DAF vs 75 DAF	5,469	2,734	2,735	DESeq2 padj<0.05 |log2FoldChange|>1
135 DAF vs 75 DAF	10,987	5,128	5,859	DESeq2 padj<0.05 |log2FoldChange|>1
170 DAF vs-75 DAF	18,168	7,812	10,356	DESeq2 padj<0.05 |log2FoldChange|>1
195 DAF vs 75 DAF	20,668	9,372	11,296	DESeq2 padj<0.05 |log2FoldChange|>1
135 DAF vs 112 DAF	1,214	677	537	DESeq2 padj<0.05 |log2FoldChange|>1
170 DAF vs 112 DAF	8,563	3,180	5,383	DESeq2 padj<0.05 |log2FoldChange|>1
195 DAF vs 112 DAF	11,821	4,642	7,179	DESeq2 padj<0.05 |log2FoldChange|>1
170 DAF vs 135 DAF	6,143	2,090	4,053	DESeq2 padj<0.05 |log2FoldChange|>1
195 DAF vs 135 DAF	11,142	4,598	6,544	DESeq2 padj<0.05 |log2FoldChange|>1
195 DAF vs 170 DAF	1,687	563	1,124	DESeq2 padj<0.05 |log2FoldChange|>1

Table S6

The KEGG annotation of DEGs.

X1st.level.pathway.	X2nd.level.pathway.	X3rd.level.pathway.	KEGG	Gene number
Metabolism	Carbohydrate metabolism	Glycolysis / Gluconeogenesis	ko03010	366
Metabolism	Carbohydrate metabolism	Citrate cycle (TCA cycle)	ko01200	272
Metabolism	Carbohydrate metabolism	Pentose phosphate pathway	ko04075	190
Metabolism	Carbohydrate metabolism	Pentose and glucuronate interconversions	ko04141	149
Metabolism	Carbohydrate metabolism	Fructose and mannose metabolism	ko04626	138
Metabolism	Carbohydrate metabolism	Galactose metabolism	ko00500	135
Metabolism	Carbohydrate metabolism	Ascorbate and aldarate metabolism	ko04064	129
Metabolism	Lipid metabolism	Fatty acid biosynthesis	ko01230	123
Metabolism	Lipid metabolism	Fatty acid elongation	ko03040	122
Metabolism	Lipid metabolism	Cutin, suberine and wax biosynthesis	ko04016	122
Metabolism	Lipid metabolism	Steroid biosynthesis	ko03013	118
Metabolism	Metabolism of cofactors and vitamins	Ubiquinone and other terpenoid-quinone biosynthesis	ko00190	101
Metabolism	Lipid metabolism	Steroid hormone biosynthesis	ko04144	100
Metabolism	Energy metabolism	Oxidative phosphorylation	ko04120	95
Metabolism	Energy metabolism	Photosynthesis	ko00230	88
Metabolism	Energy metabolism	Photosynthesis - antenna proteins	ko00520	88
Metabolism	Nucleotide metabolism	Purine metabolism	ko03018	80
Metabolism	Nucleotide metabolism	Pyrimidine metabolism	ko01212	77
Metabolism	Amino acid metabolism	Alanine, aspartate and glutamate metabolism	ko03015	74
Metabolism	Amino acid metabolism	Glycine, serine and threonine metabolism	ko00940	72
Metabolism	Amino acid metabolism	Cysteine and methionine metabolism	ko00040	70
Metabolism	Amino acid metabolism	Valine, leucine and isoleucine degradation	ko00564	70
Metabolism	Amino acid metabolism	Lysine degradation	ko00561	67
Metabolism	Amino acid metabolism	Arginine and proline metabolism	ko03030	61
Metabolism	Amino acid metabolism	Histidine metabolism	ko03050	60
Metabolism	Amino acid metabolism	Tyrosine metabolism	ko00562	55
Metabolism	Amino acid metabolism	Phenylalanine metabolism	ko00901	53
Metabolism	Xenobiotics biodegradation and metabolism	Fluorobenzoate degradation	ko00195	52
Metabolism	Amino acid metabolism	Tryptophan metabolism	ko00270	52
Metabolism	Metabolism of other amino acids	beta-Alanine metabolism	ko00900	52
Metabolism	Metabolism of other amino acids	Taurine and hypotaurine metabolism	ko03008	49
Metabolism	Metabolism of other amino acids	Selenocompound metabolism	ko00052	45
Metabolism	Metabolism of other amino acids	Cyanoamino acid metabolism	ko00240	42
Metabolism	Metabolism of other amino acids	Glutathione metabolism	ko00860	42
Metabolism	Carbohydrate metabolism	Starch and sucrose metabolism	ko03060	42
Metabolism	Glycan biosynthesis and metabolism	N-Glycan biosynthesis	ko00100	39
Metabolism	Glycan biosynthesis and metabolism	Other glycan degradation	ko04712	39
Metabolism	Glycan biosynthesis and metabolism	Various types of N-glycan biosynthesis	ko03022	37
Metabolism	Glycan biosynthesis and metabolism	Other types of O-glycan biosynthesis	ko04146	36
Metabolism	Carbohydrate metabolism	Amino sugar and nucleotide sugar metabolism	ko00970	35
Metabolism	Glycan biosynthesis and metabolism	Glycosaminoglycan degradation	ko00053	34
Metabolism	Glycan biosynthesis and metabolism	Lipopolysaccharide biosynthesis	ko02010	33
Metabolism	Glycan biosynthesis and metabolism	Peptidoglycan biosynthesis	ko04145	33
Metabolism	Lipid metabolism	Glycerolipid metabolism	ko00010	32
Metabolism	Carbohydrate metabolism	Inositol phosphate metabolism	ko00051	31
Metabolism	Glycan biosynthesis and metabolism	Glycosylphosphatidylinositol (GPI)-anchor biosynthesis	ko00760	30
Metabolism	Lipid metabolism	Glycerophospholipid metabolism	ko04020	30
Metabolism	Lipid metabolism	Arachidonic acid metabolism	ko04068	30
Metabolism	Lipid metabolism	Linoleic acid metabolism	ko00480	29
Metabolism	Lipid metabolism	alpha-Linolenic acid metabolism	ko00510	29
Metabolism	Lipid metabolism	Sphingolipid metabolism	ko00906	29
Metabolism	Glycan biosynthesis and metabolism	Glycosphingolipid biosynthesis - lacto and neolacto series	ko04014	29
Metabolism	Carbohydrate metabolism	Pyruvate metabolism	ko04110	29
Metabolism	Carbohydrate metabolism	Glyoxylate and dicarboxylate metabolism	ko04152	29
Metabolism	Carbohydrate metabolism	Propanoate metabolism	ko04371	29
Metabolism	Carbohydrate metabolism	Butanoate metabolism	ko05203	29
Metabolism	Metabolism of cofactors and vitamins	One carbon pool by folate	ko03460	28
Metabolism	Metabolism of cofactors and vitamins	Thiamine metabolism	ko00908	27
Metabolism	Metabolism of cofactors and vitamins	Riboflavin metabolism	ko03440	27
Metabolism	Metabolism of cofactors and vitamins	Vitamin B6 metabolism	ko00062	26
Metabolism	Metabolism of cofactors and vitamins	Nicotinate and nicotinamide metabolism	ko00073	25
Metabolism	Metabolism of cofactors and vitamins	Pantothenate and CoA biosynthesis	ko01210	25
Metabolism	Metabolism of cofactors and vitamins	Biotin metabolism	ko03320	25
Metabolism	Metabolism of cofactors and vitamins	Lipoic acid metabolism	ko04130	25
Metabolism	Metabolism of cofactors and vitamins	Folate biosynthesis	ko04138	24
Metabolism	Metabolism of cofactors and vitamins	Porphyrin and chlorophyll metabolism	ko04910	24
Metabolism	Metabolism of terpenoids and polyketides	Terpenoid backbone biosynthesis	ko00130	23
Metabolism	Biosynthesis of other secondary metabolites	Indole alkaloid biosynthesis	ko00330	23
Metabolism	Metabolism of terpenoids and polyketides	Monoterpenoid biosynthesis	ko00514	23
Metabolism	Metabolism of terpenoids and polyketides	Diterpenoid biosynthesis	ko04612	23
Metabolism	Metabolism of terpenoids and polyketides	Brassinosteroid biosynthesis	ko00600	22
Metabolism	Metabolism of terpenoids and polyketides	Carotenoid biosynthesis	ko00920	22
Metabolism	Metabolism of terpenoids and polyketides	Zeatin biosynthesis	ko04140	22
Metabolism	Metabolism of terpenoids and polyketides	Sesquiterpenoid and triterpenoid biosynthesis	ko04142	21
Metabolism	Energy metabolism	Nitrogen metabolism	ko05202	21
Metabolism	Energy metabolism	Sulfur metabolism	ko00730	19
Metabolism	Biosynthesis of other secondary metabolites	Phenylpropanoid biosynthesis	ko04112	18
Metabolism	Biosynthesis of other secondary metabolites	Flavonoid biosynthesis	ko04310	17
Metabolism	Biosynthesis of other secondary metabolites	Flavone and flavonol biosynthesis	ko00196	16
Metabolism	Biosynthesis of other secondary metabolites	Stilbenoid, diarylheptanoid and gingerol biosynthesis	ko00310	16
Metabolism	Biosynthesis of other secondary metabolites	Tropane, piperidine and pyridine alkaloid biosynthesis	ko00650	16
Metabolism	Biosynthesis of other secondary metabolites	Betalain biosynthesis	ko00909	16
Genetic Information I	Translation	Aminoacyl-tRNA biosynthesis	ko03410	16
Metabolism	Xenobiotics biodegradation and metabolism	Drug metabolism - cytochrome P450	ko04010	16
Metabolism	Global and overview maps	Carbon metabolism	ko04072	16
Metabolism	Global and overview maps	2-Oxocarboxylic acid metabolism	ko00260	15
Metabolism	Global and overview maps	Fatty acid metabolism	ko00904	15
Metabolism	Global and overview maps	Degradation of aromatic compounds	ko00910	15
Metabolism	Global and overview maps	Biosynthesis of amino acids	ko00350	14
Human Diseases	Drug resistance: Antineoplastic	Endocrine resistance	ko04011	14
Human Diseases	Drug resistance: Antineoplastic	Platinum drug resistance	ko05322	14
Environmental Inforn	Membrane transport	ABC transporters	ko00360	13
Environmental Inforn	Signal transduction	Two-component system	ko00750	13
Genetic Information I	Translation	Ribosome biogenesis in eukaryotes	ko04012	13
Genetic Information I	Translation	Ribosome	ko04621	13
Genetic Information I	Transcription	Basal transcription factors	ko00592	12
Genetic Information I	Replication and repair	DNA replication	ko00740	12
Genetic Information I	Transcription	Spliceosome	ko00941	12
Genetic Information I	Folding, sorting and degradation	Proteasome	ko00590	11
Genetic Information I	Folding, sorting and degradation	Protein export	ko00591	11
Organismal Systems	Endocrine system	PPAR signaling pathway	ko00902	11
Genetic Information I	Replication and repair	Base excision repair	ko00905	11
Genetic Information I	Replication and repair	Nucleotide excision repair	ko04391	11
Genetic Information I	Replication and repair	Mismatch repair	ko04810	11
Genetic Information I	Replication and repair	Homologous recombination	ko00020	10
Genetic Information I	Replication and repair	Non-homologous end-joining	ko04622	10
Genetic Information I	Replication and repair	Fanconi anemia pathway	ko04962	10
Environmental Inforn	Signal transduction	MAPK signaling pathway	ko00563	8
Environmental Inforn	Signal transduction	MAPK signaling pathway - yeast	ko00770	8
Environmental Inforn	Signal transduction	ErbB signaling pathway	ko00790	8
Environmental Inforn	Signal transduction	MAPK signaling pathway - fly	ko04340	8
Environmental Inforn	Signal transduction	Ras signaling pathway	ko04978	8
Environmental Inforn	Signal transduction	Rap1 signaling pathway	ko00513	7
Environmental Inforn	Signal transduction	MAPK signaling pathway - plant	ko00620	7
Environmental Inforn	Signal transduction	Calcium signaling pathway	ko04330	7
Environmental Inforn	Signal transduction	NF-kappa B signaling pathway	ko04614	7
Environmental Inforn	Signal transduction	FoxO signaling pathway	ko04727	7
Environmental Inforn	Signal transduction	Phospholipase D signaling pathway	ko05134	7
Environmental Inforn	Signal transduction	Plant hormone signal transduction	ko00280	6
Environmental Inforn	Signaling molecules and interaction	Neuroactive ligand-receptor interaction	ko04111	6
Cellular Processes	Cell growth and death	Cell cycle	ko04150	6
Cellular Processes	Cell growth and death	Cell cycle - yeast	ko04151	6
Cellular Processes	Cell growth and death	Cell cycle - Caulobacter	ko04350	6
Cellular Processes	Cell growth and death	Meiosis - yeast	ko04919	6
Cellular Processes	Cell growth and death	p53 signaling pathway	ko00380	5
Genetic Information I	Folding, sorting and degradation	Ubiquitin mediated proteolysis	ko00430	5
Genetic Information I	Folding, sorting and degradation	Sulfur relay system	ko00960	5
Genetic Information I	Folding, sorting and degradation	SNARE interactions in vesicular transport	ko03430	5
Cellular Processes	Transport and catabolism	Autophagy - yeast	ko04013	5
Cellular Processes	Transport and catabolism	Mitophagy - yeast	ko04015	5
Cellular Processes	Transport and catabolism	Autophagy - animal	ko04139	5
Genetic Information I	Folding, sorting and degradation	Protein processing in endoplasmic reticulum	ko04212	5
Cellular Processes	Transport and catabolism	Lysosome	ko04390	5
Cellular Processes	Transport and catabolism	Endocytosis	ko04530	5
Cellular Processes	Transport and catabolism	Phagosome	ko04974	5
Cellular Processes	Transport and catabolism	Peroxisome	ko05110	5
Environmental Inforn	Signal transduction	mTOR signaling pathway	ko05206	5
Environmental Inforn	Signal transduction	PI3K-Akt signaling pathway	ko00540	4
Environmental Inforn	Signal transduction	AMPK signaling pathway	ko01220	4
Organismal Systems	Aging	Longevity regulating pathway - worm	ko04915	4
Organismal Systems	Aging	Longevity regulating pathway - multiple species	ko05014	4
Cellular Processes	Cell growth and death	Apoptosis - fly	ko05162	4
Environmental Inforn	Signal transduction	Wnt signaling pathway	ko00640	3
Organismal Systems	Development	Dorso-ventral axis formation	ko00780	3
Environmental Inforn	Signal transduction	Notch signaling pathway	ko00785	3
Environmental Inforn	Signal transduction	Hedgehog signaling pathway	ko00945	3
Environmental Inforn	Signal transduction	Hedgehog signaling pathway - fly	ko04213	3
Environmental Inforn	Signal transduction	TGF-beta signaling pathway	ko04214	3
Environmental Inforn	Signal transduction	Apelin signaling pathway	ko04341	3
Environmental Inforn	Signal transduction	Hippo signaling pathway	ko04624	3
Environmental Inforn	Signal transduction	Hippo signaling pathway - fly	ko04911	3
Cellular Processes	Cellular community - eukaryotes	Focal adhesion	ko05010	3
Cellular Processes	Cellular community - eukaryotes	Adherens junction	ko05146	3
Cellular Processes	Cellular community - eukaryotes	Tight junction	ko05168	3
Cellular Processes	Cellular community - eukaryotes	Signaling pathways regulating pluripotency of stem cells	ko00061	2
Organismal Systems	Immune system	Antigen processing and presentation	ko00140	2
Organismal Systems	Endocrine system	Renin-angiotensin system	ko00364	2
Organismal Systems	Immune system	NOD-like receptor signaling pathway	ko00450	2
Organismal Systems	Immune system	RIG-I-like receptor signaling pathway	ko00460	2
Organismal Systems	Immune system	Toll and Imd signaling pathway	ko00550	2
Organismal Systems	Environmental adaptation	Plant-pathogen interaction	ko00630	2
Environmental Inforn	Signal transduction	TNF signaling pathway	ko00965	2
Organismal Systems	Environmental adaptation	Circadian rhythm - plant	ko00982	2
Organismal Systems	Nervous system	Synaptic vesicle cycle	ko01524	2
Organismal Systems	Nervous system	GABAergic synapse	ko03450	2
Cellular Processes	Cell motility	Regulation of actin cytoskeleton	ko04122	2
Organismal Systems	Endocrine system	Insulin signaling pathway	ko04550	2
Organismal Systems	Endocrine system	Insulin secretion	ko04668	2
Organismal Systems	Endocrine system	Estrogen signaling pathway	ko04922	2
Organismal Systems	Endocrine system	Thyroid hormone signaling pathway	ko05020	2
Organismal Systems	Endocrine system	Glucagon signaling pathway	ko05130	2
Organismal Systems	Excretory system	Vasopressin-regulated water reabsorption	ko05166	2
Organismal Systems	Digestive system	Carbohydrate digestion and absorption	ko05230	2
Organismal Systems	Digestive system	Protein digestion and absorption	ko05231	2
Organismal Systems	Digestive system	Vitamin digestion and absorption	ko00030	1
Organismal Systems	Digestive system	Mineral absorption	ko00340	1
Human Diseases	Neurodegenerative diseases	Alzheimer's disease	ko00410	1
Human Diseases	Neurodegenerative diseases	Amyotrophic lateral sclerosis (ALS)	ko00601	1
Human Diseases	Neurodegenerative diseases	Prion diseases	ko00670	1
Human Diseases	Substance dependence	Alcoholism	ko00944	1
Human Diseases	Infectious diseases: Bacterial	Vibrio cholerae infection	ko01522	1
Human Diseases	Infectious diseases: Bacterial	Pathogenic Escherichia coli infection	ko02020	1
Human Diseases	Infectious diseases: Bacterial	Legionellosis	ko03420	1
Human Diseases	Infectious diseases: Parasitic	Leishmaniasis	ko04080	1
Human Diseases	Infectious diseases: Parasitic	Amoebiasis	ko04113	1
Human Diseases	Infectious diseases: Viral	Measles	ko04115	1
Human Diseases	Infectious diseases: Viral	Influenza A	ko04320	1
Human Diseases	Infectious diseases: Viral	HTLV-I infection	ko04510	1
Human Diseases	Infectious diseases: Viral	Herpes simplex infection	ko04520	1
Human Diseases	Cancers: Overview	Transcriptional misregulation in cancer	ko04721	1
Human Diseases	Cancers: Overview	Viral carcinogenesis	ko04973	1
Human Diseases	Cancers: Overview	MicroRNAs in cancer	ko04977	1
Human Diseases	Cancers: Overview	Central carbon metabolism in cancer	ko05034	1
Human Diseases	Cancers: Overview	Choline metabolism in cancer	ko05140	1
Human Diseases	Immune diseases	Systemic lupus erythematosus	ko05164	1

Table S7

The person correlation betwwen

Gene ID	Person	Hydroxysalidroside	Hydroxytyrosol	Salidroside	7-β-1-D-glucopyranosyl-11-methyl oleoside	Tyrosol	Oleoside-11-methyl ester methyloleoside	Neonuezhenide	Ligustroflavone	Spenuezhenide	Oleuropein	Nuezhenoside G13	Ligstroside
Cluster-13715.33948	**SQS**	0.257142857	0.885714286	0.771428571	0.828571429	0.485714286	-0.657142857	1	0.885714286	0.028571429	0.771428571	-0.6	0.542857143
Cluster-13715.40847	**SQE**	0.485714286	0.942857143	0.885714286	0.771428571	0.6	-0.771428571	0.942857143	0.771428571	0.085714286	0.828571429	-0.485714286	0.6
Cluster-13715.44411	**BAS**	0.6	0.885714286	0.942857143	0.6	0.485714286	-0.657142857	0.828571429	0.542857143	-0.028571429	0.714285714	-0.428571429	0.371428571
Cluster-13715.48104	**CYP716A**	0.485714286	0.771428571	0.885714286	0.485714286	0.257142857	-0.428571429	0.771428571	0.428571429	-0.2	0.542857143	-0.485714286	0.085714286
Cluster-13715.41400	**T8G**	-0.314285714	0.428571429	0.314285714	0.657142857	-0.085714286	-0.085714286	0.771428571	0.771428571	-0.371428571	0.428571429	-0.714285714	0.085714286
Cluster-13715.46493	**GES**	0.6	1	0.942857143	0.714285714	0.714285714	-0.885714286	0.885714286	0.657142857	0.314285714	0.771428571	-0.428571429	0.657142857
Cluster-13715.50795	G10H_1	0.6	1	0.942857143	0.714285714	0.714285714	-0.885714286	0.885714286	0.657142857	0.314285714	0.771428571	-0.428571429	0.657142857
Cluster-13715.49028	G10H_2	-0.314285714	0.085714286	-0.028571429	0.2	-0.085714286	-0.085714286	0.085714286	0.085714286	0.2	-0.028571429	-0.371428571	0.085714286
Cluster-13715.22699	**IO_1**	0.942857143	0.485714286	0.542857143	-0.142857143	0.828571429	-0.542857143	0.2	-0.028571429	0.657142857	0.085714286	0.542857143	0.314285714
Cluster-13715.41846	IO_2	0.771428571	0.714285714	0.6	0.257142857	1	-0.828571429	0.485714286	0.371428571	0.828571429	0.371428571	0.257142857	0.714285714
Cluster-13715.36196	IO_3	0.942857143	0.485714286	0.542857143	-0.142857143	0.828571429	-0.542857143	0.2	-0.028571429	0.657142857	0.085714286	0.542857143	0.314285714
Cluster-13715.43322	**10GHO_1**	0.142857143	0.771428571	0.714285714	0.714285714	0.257142857	-0.428571429	0.942857143	0.771428571	-0.142857143	0.6	-0.657142857	0.257142857
Cluster-13715.59974	10GHO_2	-0.714285714	-0.314285714	-0.371428571	0.2	-0.6	0.542857143	0.142857143	0.371428571	-0.771428571	0.028571429	-0.371428571	-0.314285714
Cluster-13715.46422	10GHO_3	0.428571429	0.828571429	0.714285714	0.714285714	0.657142857	-0.714285714	0.885714286	0.828571429	0.142857143	0.771428571	-0.314285714	0.657142857
Cluster-13715.44146	10GHO_4	-0.371428571	0.314285714	0.085714286	0.828571429	0.085714286	-0.371428571	0.542857143	0.828571429	-0.257142857	0.771428571	-0.6	0.657142857
Cluster-13715.18710	10GHO_5	0.257142857	-0.371428571	-0.428571429	-0.657142857	0.371428571	0.142857143	-0.485714286	-0.371428571	0.657142857	-0.6	0.942857143	-0.028571429
Cluster-13715.48693	10GHO_6	0.428571429	0.828571429	0.714285714	0.714285714	0.657142857	-0.714285714	0.885714286	0.828571429	0.142857143	0.771428571	-0.314285714	0.657142857
Cluster-13715.43306	10GHO_7	0.428571429	0.885714286	0.828571429	0.828571429	0.542857143	-0.828571429	0.828571429	0.714285714	0.028571429	0.942857143	-0.542857143	0.714285714
Cluster-13715.50819	ISY_1	0.6	0.885714286	0.771428571	0.657142857	0.828571429	-1	0.657142857	0.542857143	0.542857143	0.771428571	-0.257142857	0.885714286
Cluster-13715.47315	ISY_2	0.6	1	0.942857143	0.714285714	0.714285714	-0.885714286	0.885714286	0.657142857	0.314285714	0.771428571	-0.428571429	0.657142857
Cluster-13715.29740	7DLGT_1	0.428571429	-0.028571429	0.142857143	-0.485714286	0.085714286	0.314285714	0.028571429	-0.2	-0.028571429	-0.428571429	0.485714286	-0.542857143
Cluster-13715.46027	7DLGT_2	0.371428571	0.942857143	0.828571429	0.771428571	0.6	-0.771428571	0.942857143	0.771428571	0.257142857	0.714285714	-0.542857143	0.6
Cluster-13715.33613	7DLGT_3	0.428571429	-0.028571429	0.142857143	-0.485714286	0.085714286	0.314285714	0.028571429	-0.2	-0.028571429	-0.428571429	0.485714286	-0.542857143
Cluster-13715.28177	7DLGT_4	0.828571429	0.428571429	0.485714286	-0.085714286	0.714285714	-0.428571429	0.257142857	0.085714286	0.428571429	0.142857143	0.485714286	0.257142857
Cluster-13715.21634	LAMT_1	0.463816829	0.927633657	0.869656553	0.81167945	0.579771036	-0.81167945	0.898645105	0.753702346	0.057977104	0.898645105	-0.521793932	0.666736691
Cluster-13715.43848	LAMT_2	0.657142857	0.942857143	0.828571429	0.6	0.885714286	-0.942857143	0.771428571	0.6	0.6	0.657142857	-0.2	0.771428571
Cluster-13715.53022	7DLH	0.485714286	0.942857143	0.885714286	0.771428571	0.6	-0.771428571	0.942857143	0.771428571	0.085714286	0.828571429	-0.485714286	0.6
Cluster-13715.44762	SLS	0.6	1	0.942857143	0.714285714	0.714285714	-0.885714286	0.885714286	0.657142857	0.314285714	0.771428571	-0.428571429	0.657142857
Cluster-13715.42925	SXS/OMES	0.828571429	0.828571429	0.771428571	0.314285714	0.942857143	-0.885714286	0.542857143	0.314285714	0.771428571	0.428571429	0.085714286	0.657142857
